# Prognostic factors of depression and depressive symptoms after hip fracture surgery: systematic review

**DOI:** 10.1186/s12877-021-02514-1

**Published:** 2021-10-10

**Authors:** R. Milton-Cole, S. Ayis, K. Lambe, M. D. L. O’Connell, C. Sackley, K. J. Sheehan

**Affiliations:** 1 Department of Population Health Sciences, King’s College London, School of Population Health and Environmental Sciences, Guy’s Campus, London, SE1 1UL UK; 2 Faculty of Medicine and Health Sciences, University of Nottingham, Nottingham, UK

**Keywords:** Hip fracture, Depression, Prognostic factors, Predictors

## Abstract

**Background:**

Patients with hip fracture and depression are less likely to recover functional ability. This review sought to identify prognostic factors of depression or depressive symptoms up to 1 year after hip fracture surgery in adults. This review also sought to describe proposed underlying mechanisms for their association with depression or depressive symptoms.

**Methods:**

We searched for published (MEDLINE, Embase, PsychInfo, CINAHL and Web of Science Core Collection) and unpublished (OpenGrey, Greynet, BASE, conference proceedings) studies. We did not impose any date, geographical, or language limitations. Screening (Covidence), extraction (Checklist for critical Appraisal and data extraction for systematic Reviews of prediction Modelling Studies, adapted for use with prognostic factors studies Checklist), and quality appraisal (Quality in Prognosis Studies tool) were completed in duplicate. Results were summarised narratively.

**Results:**

In total, 37 prognostic factors were identified from 12 studies included in this review. The quality of the underlying evidence was poor, with all studies at high risk of bias in at least one domain. Most factors did not have a proposed mechanism for the association. Where factors were investigated by more than one study, the evidence was often conflicting.

**Conclusion:**

Due to conflicting and low quality of available evidence it is not possible to make clinical recommendations based on factors prognostic of depression or depressive symptoms after hip fracture. Further high-quality research investigating prognostic factors is warranted to inform future intervention and/or stratified approaches to care after hip fracture.

**Trial registration:**

Prospero registration: CRD42019138690.

**Supplementary Information:**

The online version contains supplementary material available at 10.1186/s12877-021-02514-1.

## Introduction

Hip fractures are among the most common orthopaedic injuries affecting 66,313 older adults in the United Kingdom in 2018 [1]. These fractures can negatively affect patients’ health-related quality of life as they often lead to losses in mobility and independence [2], a need for ongoing care, limitations in activities of daily living (ADLs) [3] and ensuing death [4]. To mitigate these risks, prompt surgery and a subsequent period of rehabilitation is the definitive management approach for most hip fractures [5].

Psychiatric illness, including depression and depressive symptoms, is common in the population of older adults with hip fracture [6]. The reported prevalence of depression among patients with hip fracture is between 9 and 47%, depending on the country, population, duration of depressive symptoms, the method used to assess depression and the type of hip fracture [7, 8]. Patients with depression or depressive symptoms are less likely to recover functioning (as measured by function, e.g., balance or walking speed, and activities, e.g., activities of daily living) after hip fracture compared to those without depression [9]. The risk of developing depression or depressive symptoms is highest before discharge and in the 12 months following the event [10]. Indeed, Maharlouei and colleagues reported depression as a major contributing prognostic factor for recovery after hip fracture [11] and those with consistently high levels of depressive symptoms following hip fracture were at a considerably increased risk of not returning to their baseline physical function [12].

Several studies have identified prognostic factors for depression or depressive symptoms after hip fracture [13–15]. These prognostic factor studies investigate which characteristics are associated with changes in depressive symptoms or the occurrence of new-onset depression [16]. The purpose of such studies is to gain a better understanding of the disease process and to define risk groups based on outcome prognosis [17]. This would enable the development of new interventions or quality improvement initiatives targeting modifiable prognostic factors and/or stratified approaches to care for non-modifiable prognostic factors [16] . In addition, while a given factor may not be modifiable, the proposed underlying mechanisms for its association with the outcome may be modifiable [18], further informing interventions and quality improvement initiatives.

To date, there has been no attempt to synthesise the evidence on prognostic factors of depression or depressive symptoms after hip fracture or to assess the underlying mechanisms for the association between these factors and depression or depressive symptoms. This is important as an understanding of the extent and nature of prognostic factors of depression or depressive symptoms could help to inform future approaches to optimise recovery after hip fracture. Therefore, this review aims to identify prognostic factors of depression or depressive symptoms up to 1 year after hip fracture surgery in adult patients. The secondary aim is to summarise the proposed underlying mechanisms for their association with depression or depressive symptoms.

## Methods

The protocol for this systematic review is registered on the International Prospective Register of Systematic Reviews (PROSPERO: CRD42019138690).

### Eligibility criteria

This review included studies of prognostic factors for depression or depressive symptoms in adults over the age of 18 who have undergone surgery for a non-pathological hip fracture. We adopted an inclusive approach to eligibility with exclusions limited to children (those below the age of 18 years), those treated for a pathological fracture, and those treated conservatively. No geographical, language or date limits were applied.

### Search strategy

Databases were searched to identify relevant published (MEDLINE, Embase, PsychInfo, CINAHL, Web of Science Core Collection) and unpublished (OpenGrey, Greynet and Bielefeld Academic Search Engine (BASE) and conference proceedings) studies from inception to 9th November 2020. Reference lists of included studies were reviewed for any further relevant studies. The search strategy was developed using previously published search terms and synonyms identified in the search strategies of published Cochrane reviews conducted on hip fractures and depression [19–22], terminology used in NICE guidelines, through the MeSH database and Ovid MEDLINE subject heading function. The search strategy also included the published recommended search strategy for identifying prognostic factor studies (Supplementary File 1) [23].

### Study selection

References were exported into Covidence for deduplication and screening [24]. Two reviewers screened the titles and abstracts independently and then carried out full-text screening against the eligibility criteria. Conflicts were resolved by consensus or by a third author if consensus could not be reached.

### Data extraction

Three authors extracted data for all included studies independently using the modified Checklist for critical Appraisal and data extraction for systematic Reviews of prediction Modelling Studies, adapted for use with prognostic factors studies (CHARMS-PF checklist) [25]. Information extracted included the authors’ names, publication year, study dates, setting and design, timepoints definitions, outcomes, prognostic factors information, sample size, analysis methods, and results data. Data on the proposed underlying mechanisms for reported associations were also extracted. Any disagreements during this stage were resolved by consensus. If any information was missing or incomplete, an attempt was made to contact the study authors to retrieve the missing data.

### Quality appraisal

Three authors assessed the methodological quality of all included studies independently using the refined QUality In Prognosis Studies (QUIPS) appraisal tool. QUIPS is a six-domain checklist used to assess risk of bias in prognostic factor studies (at the study level) [26]. The domains are study participation, study attrition, prognostic factor measurement, outcome measurement, study confounding and statistical analysis and reporting. Grading of each domain consisted of three options: high, moderate, or low risk of bias. Disagreements in risk of bias judgements were resolved by consensus.

### Analysis

We evaluated the data extraction table for homogeneity between studies. There was substantial heterogeneity in study design, prognostic factors investigated, and their methods of measurements, study timepoints, methods of analysis and reporting of results. Therefore, conducting a meta-analysis was not possible. We reported the results in a narrative synthesis using text, figures, and tables [27]. We organised factors according to whether they related to the patient or care structures and processes, and their proposed underlying mechanisms.

## Results

### Study selection

**Fig. 1:**
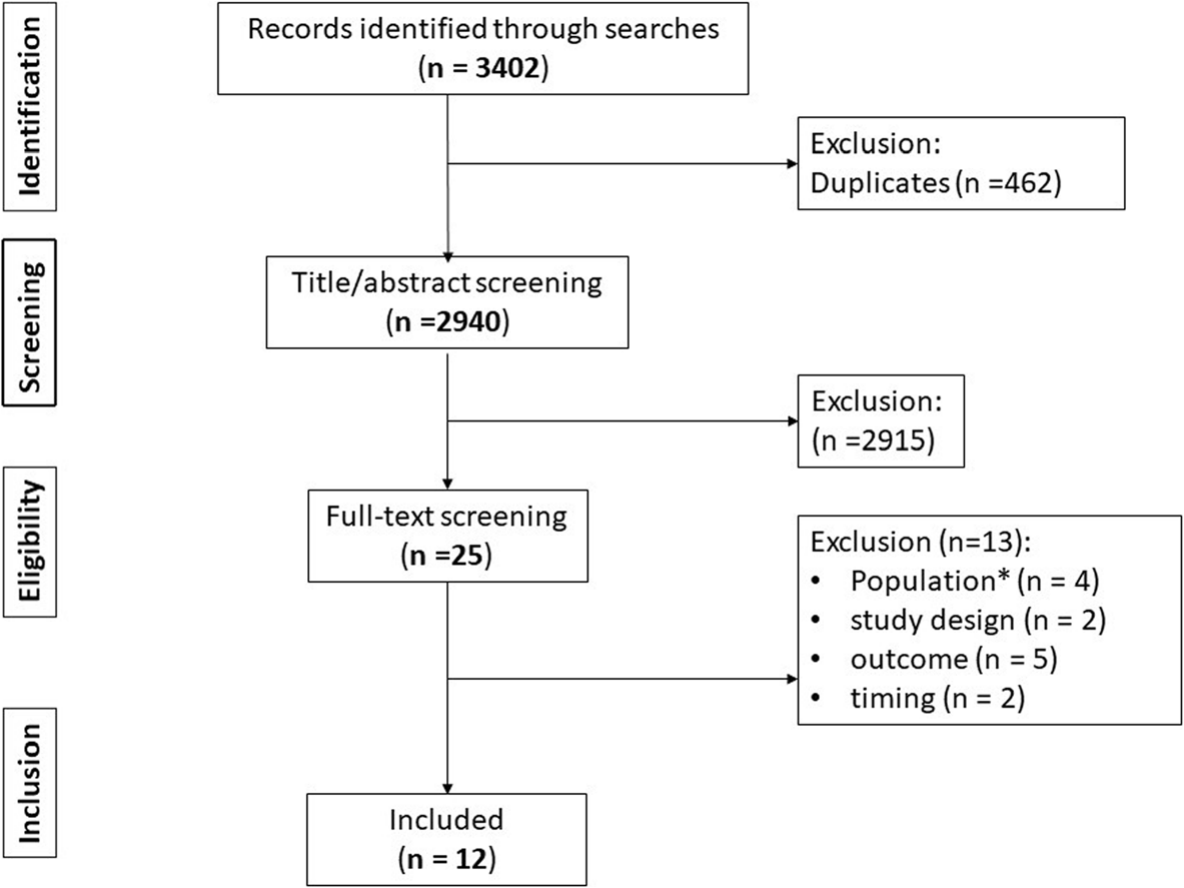
PRISMA Flowchart. * Control groups without hip fracture and analysis limited to comparisons between those with and without hip fracture *n* = 2; participants included those with fractures of any limb *n* = 2

We identified 3402 studies from five databases, 462 of which were duplicates. During title/abstract and full-text screening, 2915 studies were excluded. On full text review we excluded studies by population where control groups who had not suffered a hip fracture were used and the results were limited to comparisons between those with and without hip fracture and where the sample included any patient who sustained fall-related injuries to any limb (*n* = 4), study design as the level of depression in patients with cognitive impairments was investigated rather than the prognostic factors of depression or depressive symptoms and the study was of cross-sectional design (*n* = 2), outcome; where outcomes included the effect of ageing on immunity, the prognosis of mental disorders, the impact and cause of depression after hip fracture, functional recovery and the prognosis of outcomes in those with depression after hip fracture (*n* = 5), and timing when follow up times were up to 2 years after discharge and 3 years after hospitalisation (*n* = 2). Therefore, 12 studies were included in this review. Study selection is summarised in Fig. 1.

### Study characteristics and measures of depression

Characteristics of each study are summarised in Table 1 below. This review included 12 studies consisting of 2642 patients. The sample size ranged from 23 [32] to 570 [14], with a median sample size of 146. The mean age of patients ranged from 76.2 [32] - 81.8 [13] years. Depression or depressive symptoms were measured using the Depression Anxiety Stress Scale in one study [28], the Montgomery- Åsberg Depression Rating Scale in three studies [15, 30, 35], Structured Clinical Interview for DSM-IV disorders (SCID-IV) in two studies [15, 32], the Geriatric Depression Scale – Short Form (GDS-SF) in one study [29], the Geriatric Depression Scale in three studies [31, 34, 35], the Geriatric Depression Scale-Chinese version in two studies [10, 33], the Hamilton Depression Rating Scale (Ham-D) in two studies [13, 32], Primary Care Evaluation of Mental Disorders in one study [13] and the Hospital Anxiety and Depression Scale (HADS) in two studies [14, 35]. Baseline timepoints (where reported) ranged from on admission [29] to prior to discharge [10, 13, 32, 33] or 22 days post-hip fracture [34]. Follow up time points ranged from 1-week post-surgery [30] to 12 months after hip fracture/surgery [10, 14, 30, 31, 33, 34].

**Table 1:** Study characteristics

Author, year	Sample size	Population Characteristics	Prognostic Factors	Outcome Measures	Study Timepoints
Bruggeman 2007, Australia [28]	103	78 years 79% female	Personal control, acute stress, illness perception, pain, physical mobility	Depression Anxiety Stress Scale	Study dates not stated Baseline = Within one week of surgery Followed up at 3-weeks
Cristancho 2016, USA [15]	430	78.2 years 75.8% female 93.7% Caucasian 22.4% clinically diagnosed with new onset depression	Age, gender, medical illness burden, antidepressant use, smoking history, pain ratings, cognitive status, functional recovery, stress ratings, social support, anxiety, history of minor/major depression, implant type	Montgomery- Åsberg Depression Rating Scale, Structured Clinical Interview for DSM-IV disorders (SCID-IV)	2008–2012 Baseline = approx. 2 days post-surgery Followed up at 1, 2, 4, 8, 12, 26, and 52 weeks after baseline
Deng, 2005, Taiwan [29]	146	77.7 years > 90% unemployed	Gender, Cognition, Residence status, Prefracture physical function, support system, age, marital status, religion, occupation, diseases before admission	Geriatric Depression Scale – Short Form (GDS-SF)	2001–2003 Baseline = On admission Followed up at 1 month after discharge
Langer 2015, USA [30]	500	78.4 years 73.8% women 92.5% white	Positive and negative affect, age, education, gender and social support, chronic illness	The Montgomery Åsberg Depression Rating Scale - 7-day version	Not stated Baseline = 2 to 7 days post-surgery Followed up at Week 1, Week 2, Week 4, Week 8, Week 12, Week 26, and Week 52 post surgery.
Lenze, 2007, USA [13]	126	Major Depressive Disorder group: 78.3 years 16.7% male 100% Caucasian No Major Depressive Disorder group: 81.8 years 20.4% male 91.7% Caucasian 61% MDD cases had onset of MDD symptoms before discharge from the acute care hospital, whereas the other seven had onset of symptoms between 2 and 10 weeks after the hospitalization	Demographic variables and characteristics of the fracture, Functional status, Delirium, cognition, social support, medical comorbidity, Apathy, memory, executive function, Surgery LOS, characteristics of the surgery	Hamilton Depression Rating Scale (Ham-D), Primary Care Evaluation of Mental Disorders	2002–2004 Baseline = End of hospital stay. Followed up after 2 weeks, then every 4 weeks until 26 weeks post hospital discharge Functional status measured prefracture through interview during hospital stay
Lenze, 2008, USA [31]	145	81.2 years 95.9% Caucasian woman	Pre-fracture function, comorbidities, ADLs, genetic samples-molecular weight DNA, 5HTR1A and 5HTR2AA	Geriatric Depression Scale	1998–2004 Baseline = 12 days post-fracture Followed up at 2, 6, and 12-months post-fracture
Lenze, 2005, USA [32]	23	s Allele group: 78.8 years 77% female 100% white l/l Genotype group: 76.2 years 100% female 90% white	Genotype −5-HTTLPR	Hamilton Rating Scale for Depression, mood module of the Structured Clinical Interview for DSM-IV Axis I Disorders	2002–2003 Baseline = end of their hospital stay Followed up at 2, 6, 10 and 14 weeks after hospital discharge
Liu 2018, Taiwan [33]	179	76.7 years 68.2% female Average number of comorbidities was 2.45	Age, gender, marital status, educational level, comorbidities, cognitive impairment, functional impairment, care model and group membership probability, prefracture mobility	Geriatric Depression Scale-Chinese version	2005–2010 Baseline = before discharge Followed up at 1, 3, 6, and 12-months post- discharge
Matheny, 2011, USA [34]	134	81.7 years 96.3% Caucasian All participants were female	Age, height, weight, comorbidities, cognitive status, inflammatory cytokines, lower extremity function	Geriatric Depression Scale (GDS)	1998–2004 Baseline-within 22 days post-hip fracture Followed up at 2, 6 and 12-months post fracture
Shyu 2009, Taiwan [10]	147	77.9 years 67.3% female 49% illiterate	Gender, age, concomitant illnesses, prefracture performance of ADLs, education, emotional-social support, and cognitive status.	Geriatric Depression Scale-Chinese version	2001–2003 Baseline = prior to discharge Followed up at 1, 3, 6 and 12 months after hospital discharge
Van der Ree, 2020, Netherlands [14]	570	78.4 years 393 females 46.3% frail 3.7% had early-onset dementia	Age, gender, ASA, Prefracture residential status, Fracture type, LOS, Discharge location, Pre-fracture health status, Pre-fracture frailty	Hospital Anxiety and Depression Scale (HADS)	2015–2016 Baseline = 1 week after hip fracture. Followed up at 1, 3, 6 and 12 months after hip fracture.
Voshaar 2007, the Netherlands [35]	139	80.7 years 77% women	Pain, fear of falling, anxiety, functional outcomes	Geriatric Depression Scale, The Montgomery Åsberg Depression Rating Scale, Hospital Anxiety and Depression Scale	Baseline = not described Followed up at six weeks, three months, and six months

### Risk of bias in studies

The quality of the studies included in this review was variable, with 10 of 12 studies at high risk of bias in at least one domain and no study at low risk of bias across all domains (Table 2). Three out of the 12 studies were at moderate risk of bias for study participation [28–30], while the other nine studies were at low risk of bias [10, 13–15, 31–35]. Five studies were at high risk of bias for study attrition [15, 28–30, 34], four studies were judged to be at moderate risk [14, 31, 33, 35], and the others deemed to be at low risk of bias [10, 13, 32]. One study [29] was judged to be at high risk of bias for prognostic factor measurement, six out of the 12 studies were at moderate risk of bias [10, 15, 28, 33–35], and the remaining five studies were at low risk [13, 14, 30–32]. One study was at high risk of bias for outcome measurement [29], with all other studies deemed at low risk. Study confounding had the highest risk of bias overall, with seven studies being high risk [10, 14, 15, 29, 30, 32, 35], two studies were at moderate risk of bias [28, 33] and three studies at low risk [13, 31, 34]. Statistical analysis and reporting were at high risk of bias in five studies [10, 29, 32–34], moderate risk in five studies [13, 15, 30, 31, 35] and two studies were judged to be at low risk of bias [14, 28]. Detailed rationale for each risk of bias assignment is presented in Supplementary File 2.

**Table 2:** Results of Quality Appraisal using the Quality In Prognosis Studies (QUIPS) Tool Summary

Author, year	Study participation	Study attrition	Prognostic factor measurement	Outcome measurement	Study confounding	Statistical analysis/reporting
Bruggeman 2007, Australia [28]	moderate	high	moderate	low	moderate	Low
Cristancho 2016, USA [15]	low	high	moderate	low	high	moderate
Deng, 2005, Taiwan [29]	low	high	high	high	high	high
Langer 2015, USA [30]	moderate	high	low	low	high	moderate
Lenze, 2007, USA [13]	low	low	low	low	moderate	moderate
Lenze, 2008, USA [31]	low	moderate	low	low	low	moderate
Lenze, 2005, USA [32]	low	low	low	low	high	high
Liu 2018, Taiwan [33]	low	moderate	moderate	low	moderate	high
Matheny, 2011, USA [34]	low	high	moderate	low	low	high
Shyu 2009, Taiwan [10]	low	low	moderate	low	high	high
Van der Ree, 2020, Netherlands [14]	low	moderate	low	low	high	low
Voshaar 2007, the Netherlands [35]	low	moderate	moderate	low	high	moderate

#### Prognostic factors

A total of 37 prognostic factors were investigated across the 12 studies included in this review (Table 3). Most studies did not identify a primary prognostic factor of interest (rather reporting on multiple factors from one model). Lenze [32] was the only study to identify a primary prognostic factor reporting a positive association between Genotype − 5-HTTLPR and depression or depressive symptoms after hip fracture. Factors explored by more than one study included age [13–15, 30], gender [13–15, 29, 30], cognitive status [13, 15, 29], comorbidities [13, 15, 31, 33], fracture type [13, 14], anxiety [15, 35], pain [15, 28, 35], residence status [14, 29], social support [13, 15, 29, 30], stress [15, 28], activities of daily living [15, 29, 31, 33], mobility [14, 15, 28, 35] and function [13, 35]. Four authors were contacted for additional information however this additional information was not available (*n* = 2), or the authors did not respond to our request (*n* = 2).

**Table 3:** Study’s Results

Author, year	Analysis	Effect Estimate/Result	Proposed Mechanism
Bruggeman 2007, Australia [28]	Hierarchical multivariable regression	Adjusted R^2^, Overall Model *F* Personal control predicting Depression: 0.43, F (4, 52) Personal control and hopelessness predicting depression: 0.55, F (5, 46) Personal control beliefs predicted depression severity when entered alone, this relationship became nonsignificant when hopelessness scores were entered Depression at T1 and pain at time T2 were significant predictors of depression	N
Cristancho 2016, USA [15]	Multinomial logistic regression model	Odds Ratio Age: 1.04 (0.98–1.10) Antidepressant use: 4.61 (1.46–14.61) Anxiety traits: 1.49 (1.25–1.78) CIRS-G co-morbidities: 1.05 (0.92–1.20) FRS mobility score: 1.02 (0.89–1.17) GALES stress rating: 1.38 (1.17–1.64) Gender 0.39 (0.12–1.26) Implant: type – internal fixation with screws: 2.75 (0.77–9.77) Implant type – sliding hip screw – IM nail, other: 7.94 (2.31–27.31) History of depression: 4.02 (1.13–14.28) Pain rating scale: 1.09 (0.92–1.30) SBT cognitive score: 1.07 (0.91–1.24) Smoking status – current: 5.11 (1.09–24.00) Smoking status – past: 1.67 (0.52–5.31) Social network: 1.09 (0.98–1.22) Subjective support: 1.39 (1.12–1.72)	N
Deng, 2005, Taiwan [29]	Logistic regression	Odds Ratio Gender: 5.486 (2.088–14.416) Complete cognition: 0.434 (0.143–1.321) Fixed residence: 0.482 (0.099–2.338) Dependent physical function before fracture: 6.021 (2.034–137.823) Supporting system: 0.981 (0.940–1.023)	Y
Langer 2015, USA [30]	Auto-regressive latent trajectory (ALT) analyses	Fully standardized parameter used (no further description given) Depression: Negative affect intercept: 0.48 Negative affect slope: 0.56 Slower decline in negative affect predicted higher depression at Week 52	Y
Lenze, 2007, USA [13]	Repeated-measures mixed-effects model; Univariable and Multivariable Logistic Regressions	Odds Ratio Univariate: Age: 0.96 (0.91–1.01) Male: 1.28 (0.34–4.81) Cumulative Illness Rating Scale score: 0.98 (0.84–1.14) Prefracture FIM motor subscale score: 1.01 (0.96–1.06) Post fracture FIM motor subscale score: 1.01 (0.96–1.05) Sub capital fracture: 0.65 (0.23–1.86) Prosthetic joint surgery: 0.76 (0.26–2.17) Surgical LOS: 1.04 (0.92–1.19) Apathy Evaluation Scale score: 1.09 (1.03–1.16) Delirium Rating Scale score: 1.07 (0.99–1.16) MMSE: 1.01 (0.88–1.15) Logical Memory Test score: 1.03 (0.89–1.19) Mattis Initiation-Perseveration scale: 0.93 (0.83–1.04) Social support: 0.97 (0.91–1.03) Multivariate: Apathy: 1.09 (1.02–1.15) Delirium: 1.05 (0.96–1.15) Individuals with clinical evidence of apathy are at high risk for developing MDD or depressive symptoms	N
Lenze, 2008, USA [31]	General linear regression models – time-adjusted model and covariate and time-adjusted model	Estimated mean difference Covariate and time-adjusted model: 5HTR1A only: 0.66 (0.18, 1.14) 5HTR2A only: 0.44 (−0.10, 0.98) 5HTR1A and 5HTR2A combined: 5HTR1A: 0.61 (0.13, 1.09) 5HTR2A: 0.37 (− 0.16, 0.91) The 5HTR1A promoter polymorphism is associated with depressive symptoms in elderly persons after a hip fracture. The G allele of the 5HTR1A (− 1019) polymorphism was associated with increased depressive symptoms for 12 months after the fracture	Y
Lenze, 2005, USA [32]	Repeated-measures analysis of variance	Only baseline data given The s allele of the 5-HTTLPR is predictive of having MDD and high depressive symptoms after hip fracture	N
Liu 2018, Taiwan [33]	Binary logistic regression modelling	Coefficient estimate/b Number of comorbidities: −0.27 (− 0.54, − 0.00) Pre-fracture mobility: 0.11 (0.04, 0.18) Other results not given Patients were more likely to be in the progressively lower-risk group than in the fluctuating higher-risk group if they had fewer comorbidities or better prefracture mobility	N
Matheny, 2011, USA [34]	Generalized estimating equations (GEE)	Not given, only *p*-values We found that hip fracture patients in the highest group of inflammatory cytokine levels for both IL-6 and sTNF-αR1 had higher levels of depressive symptoms than those in the lowest group, particularly at 12 months post fracture.	Y
Shyu 2009, Taiwan [10]	Multivariable logistic regression	Effect Estimates not given Those who were female (P < 0.001), with lower prefracture performance of ADLs (*P* < 0.001) and with lower emotional-social support (P < 0.001) were more likely to be at higher risk for depressive symptoms. Lower emotional-social support was the only predictor for persistent depressive symptoms after discharge None of the predictors in the model were statistically significantly associated with depressive symptoms after discharge.	N
Van der Ree, 2020, Netherlands [14]	Univariable and Multivariable Logistic Mixed Model	Odds Ratio Multivariable including frailty Age ≥ 80 years: 1.61 (0.87–2.99) Female gender: 0.87 (0.48–1.57) ASA III/IV/V: 1.97 (0.94–4.12) Prefracture residential status: 0.92 (0.29–2.90) Prefracture mobility: With aid: 0.89 (0.44–1.78) Dependent: 1.54 (0.46–5.13) Type of fracture: extracapsular: 1.50 (0.85–2.65) Length of hospital stay (days): 1.11 (1.04–1.20) Discharge location: Institution: 2.20 (1.12–4.34) Frailty: 2.74 (1.41–5.34) Higher ASA scores, dependence in locomotion at baseline, longer LOS at hospital, and discharge to an institution were prognostic factors for symptoms of depression during 1 year after hip fracture Frailty at onset of hip fracture was the most important prognostic factor of symptoms of depression on average in the year following hip fracture	N
Voshaar 2007, the Netherlands [35]	Cox proportional hazards model	Hazard Ratio Postoperative pain: 1.32 (1.14–1.53) Baseline anxiety: 1.25 (1.08–1.44) The independent predictors that were associated with incident depression yielded postoperative pain and baseline anxiety as the strongest, independent risk factors	N

### Patient-related factors

Twelve studies explored 35 factors relating to patient characteristics. Four factors were accompanied by a proposed underlying mechanism for their reported association.

Apathy [13], anxiety [15, 35], discharge location [14], inflammatory cytokines [34], personal control beliefs [28], current smoking status [15], negative affect [30], American Society of Anaesthesiologists (ASA) [14], history of depression [15], antidepressant use [15], and pre-fracture frailty [14] were positively associated with depression or depressive symptoms after hip fracture. Delirium [13], hopelessness [28], fear of falling [35], chronic illness [30], executive function [13], memory [13], pre-fracture health status [14], education [30] and fracture type [13, 14] were not associated with depression or depressive symptoms after hip fracture. Four studies reported no association between age and depression or depressive symptoms after hip fracture [13–15, 30]. There was no positive association reported between residence status and depression or depressive symptoms after hip fracture investigated by two studies [14, 29]. There was conflicting evidence for an association between pain and depression or depressive symptoms after hip fracture, two studies reporting a positive association [28, 35] and one study reporting no association [15]. Similarly, two studies reported conflicting evidence for a positive association between stress and depression or depressive symptoms after hip fracture [15, 28]. Three studies reported no association between lack of social support and depressive symptoms after hip fracture [13, 29, 30].

In contrast, one study reported low social support was associated with depression or depressive symptoms in hip fracture patients [15]. One study reported an association between lower performances of activities of daily living and depression or depressive symptoms after hip fracture (measured using the Chinese version of the Barthel Index) [33]. Another study reported an association between lower performances of activities of daily living and instrumental activities of daily living (measured using the Chinese version of the Barthel Index and the Instrumental activities of daily living scale (IADLs)) with depression or depressive symptoms after hip fracture [29], while two studies reported no association [15, 31]. These studies measured activities of daily living and instrumental activities of daily living using Basic activities of daily living (BADLs) and IADLs scales [15] and the Lower extremity Physical activities of daily living and IADLs scales [31] respectively. One study reported an association [14] and three reported no association [15, 28, 35] between reduced mobility and depression or depressive symptoms after hip fracture. Two studies reported no association between lower function (measured by Functional Independence Measure, gait test (the time and number of steps taken in a 4-m walk) and the functional reach test) and depression or depressive symptoms after hip fracture [13, 35].

There were further inconsistent findings for the association between gender, comorbidities and pre-fracture residence and depression or depressive symptoms after hip fracture. Four studies reported no association between gender and depression or depressive symptoms after hip fracture [13–15, 30]. In contrast, Deng reported women were five times more likely to have depression or depressive symptoms than men [29]. Four studies investigated the association between comorbidities and depressive symptoms [13, 15, 31, 33]. One study reported an association suggesting patients with more comorbidities were more likely to be in the higher risk group for depression or depressive symptoms than those with less comorbidities [33]. Three studies reported no association between a lower cognitive status and depressive symptoms [13, 15, 29]. Two studies reported an association between the presence of genotypes 5HTR1A and 5-HTTLPR [31, 32] and no association between 5HTR2A [31] and depression or depressive symptoms after hip fracture.

#### Underlying mechanisms

Most studies did not propose an underlying mechanism for the association between their prognostic factor/s of interest and depression or depressive symptoms after hip fracture. Lenze [31] reported 5HTR1A was predictive of depressive symptoms due to the interaction between genetics and social-environmental stressors. Deng [29] proposed the role shift from caregiver to care-receiver resulting in feelings of conflict between their physical ability and social expectations, as a potential mechanism for their reported association between gender and depression or depressive symptoms. Matheny [34] proposed an underlying mechanism that the increased cytokines may indicate a chronic sickness syndrome or due to the transient stimulation of these cytokines by physical and psychological stressors for their reported association between social support and depression or depressive symptoms. A proposed mechanism of the association between social support and depression or depressive symptoms after hip fracture is an extensive social network may have a protective role in times of distress [30].

### Process/structure-related factors

**Fig. 2:**
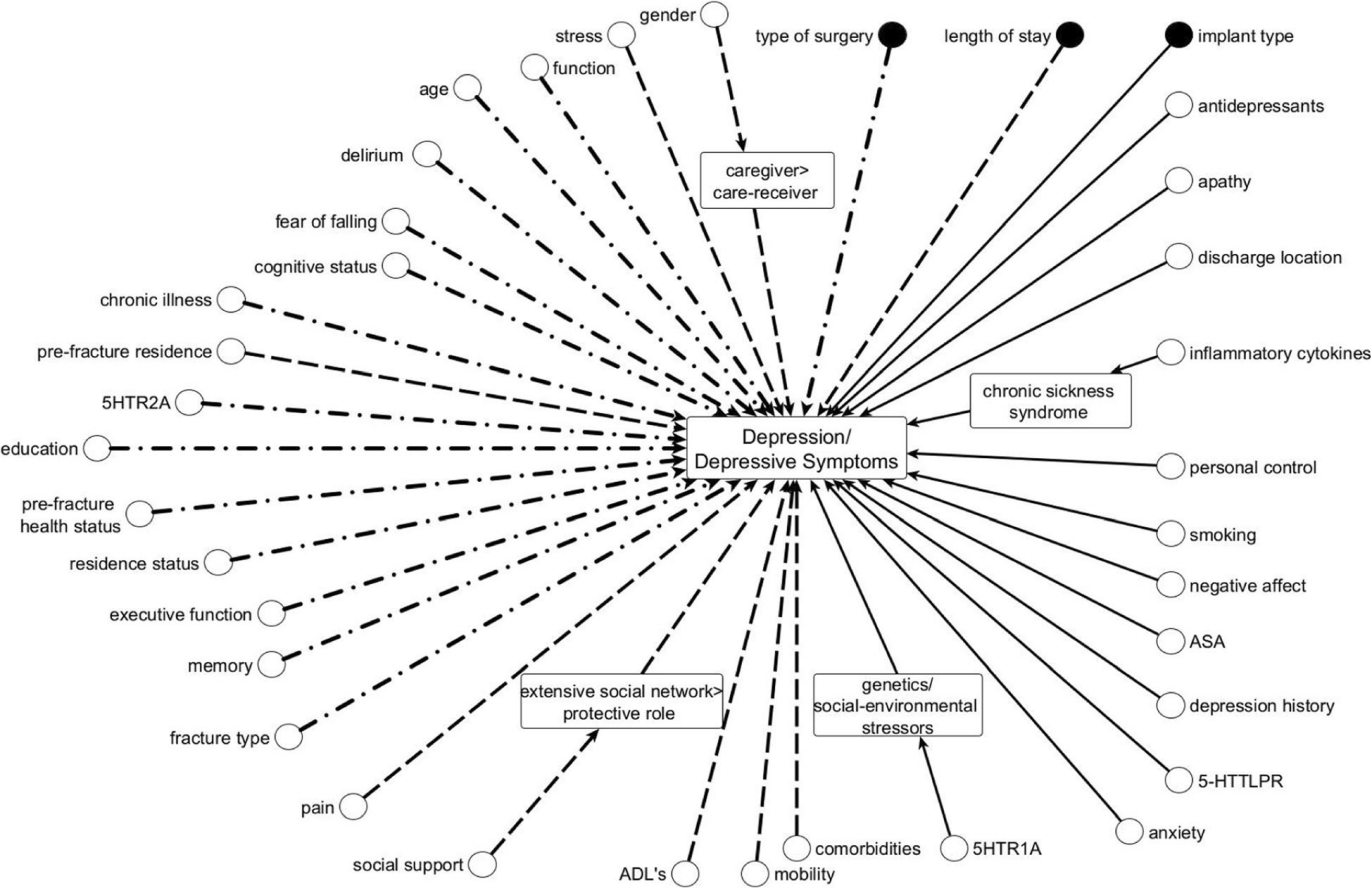
Prognostic Factors of Depression or Depressive Symptoms identified in this review. Nodes represent prognostic factors. Dashed arrows indicate conflicting evidence for the presence of an association. Straight arrows indicate a reported association. Unequal dashed arrows indicate no reported association. The rounded edge square boxes indicate reported underlying mechanisms. Black nodes denote structure/process factors

Three studies explored factors related to care processes or structures [13–15]. Two studies investigated the association between a longer length of stay [13, 14] and depression or depressive symptoms after hip fracture. Lenze [13] reported no association between length of stay and depression or depressive symptoms after hip fracture. In contrast, van de Ree [14] reported a longer length of hospital stay was associated with depression or depressive symptoms in the year after hip fracture. The type of surgery was not associated with depression or depressive symptoms in one study (39% of participants received prosthetic joint surgery, other types of surgery are not described) [13]. In contrast, in the study by Cristancho [15], implant type was associated with depression or depressive symptoms whereby patients who had a sliding hip screw and intramedullary nail were more likely to develop depression or depressive symptoms compared to patients who had an internal fixation with screws [15]. No study proposed an underlying mechanism for the association between these factors and depression or depressive symptoms after hip fracture. Figure 2 shows all prognostic factors investigated and whether an association was reported with depression or depressive symptoms.

## Discussion

We identified 37 prognostic factors of depression or depressive symptoms after hip fracture surgery across 12 studies. Most studies investigated patient factors, with only a few related to care process or structure factors. Few studies proposed an underlying mechanism for the reported association. For factors assessed by more than one study, there was often conflicting evidence which may be attributed to the heterogeneity of the studies. Synthesis of the study’s results was challenging due to heterogeneity in study design, methods of assessments for the prognostic factors and depression or depressive symptoms, time points evaluated, and the prognostic factors investigated. Therefore, identifying which prognostic factors are the strongest predictors of depression or depressive symptoms after hip fracture surgery was not possible. This was further compounded by the lack of studies explicitly defining their primary prognostic factor. This approach introduces the phenomenon known as the “Table 2 fallacy” [36], where the effect estimates of secondary prognostic factors are inappropriately interpreted alongside the estimates of the primary prognostic factor. This may lead to an overestimation of the association between these secondary prognostic factors and outcomes as models are likely insufficient to control for confounding of secondary factors associated with the outcome. Further, the prognostic factors taken into consideration are often highly correlated, and one factor may work as a proxy for another. Analysing such factors together may render a factor unimportant, yet in another study where no correlated factors were considered, the same factor may be important.

Only three studies were deemed to be at low risk of bias for attrition. An additional concern related to attrition was a failure to report the extent of missing data across several studies [13, 28, 32, 33]. Missing data can lead to invalid conclusions due to a reduction in the study’s statistical power, representativeness of the study sample, and bias in the estimations made [37]. For the current review, the rate of attrition likely increased with the severity of depressive symptoms and therefore data is likely not missing at random [38]. This presents challenges for analysis as common missing data techniques (e.g., imputation) are not appropriate for data not missing at random but the estimate of the effect may be biased due to the missingness [39]. This limits the generalizability of the prognostic factors identified to those with more severe depression/depressive symptoms.

The current review highlights a dearth of evidence investigating structures or processes of care. A longer length of stay was identified as a prognostic factor for depressive symptoms in the first year after hip fracture. It has previously been shown that depression can increase a patient’s length of hospital stay after hip fracture surgery [40]. This potentially indicates a bi-directional relationship in which experiencing depressive symptoms increases a patient’s length of stay as well as a longer length of stay being a predictor of depressive symptoms after hip fracture surgery. However, the association was disputed by Lenze [13], and therefore this relationship warrants further study. Similarly, inconsistency in the evidence for an association between surgery type and depression or depressive symptoms was reported [13, 15]. All studies failed to propose an underlying mechanism for putative associations between structures, processes, and depression or depressive symptoms. Failure to identify a plausible underlying mechanism could result in observing a statistical association in the absence of causation.

Most studies identified patient factors associated with depression or depressive symptoms after hip fracture. For example, one study reported the inflammatory cytokines interleukin-6 (IL-6) and tumour necrosis factor-alpha (sTNF-αR1) were associated with depressive symptoms 1 year after hip fracture [34]. These inflammatory markers are also associated with adverse outcomes postoperatively, such as complications [41]. These unfavourable outcomes may be what leads to depressive symptoms in hip fracture patients postoperatively. IL-6, a pro-inflammatory marker, is involved in the disease progression of osteoarthritis [42]. While no therapies targeting IL-6 pathway inhibitors in individuals with osteoarthritis have been developed, the antibody tocilizumab is an effective treatment in certain conditions when IL-6 levels are increased [42]. Future research into therapies blocking the pro-inflammatory cytokines IL-6 and sTNF-αR1 in hip fracture patients may provide an intervention which influences the development of depression or depressive symptoms after hip fracture. There were inconsistencies between studies on whether pain was associated with depression or depressive symptoms after hip fracture. This difference may be due to the variations in end points. The two studies which found an association followed-up at 3 weeks [28] and up to 6 months [35] compared to the study that did not find an association that followed up to a year after baseline [15]. Acute pain has previously been associated with depression this may explain the differences in findings [43]. We noted that some prognostic factors were modifiable and are therefore amenable to change through intervention which in turn leads to improvements in patient’s quality of care.

Most factors we identified were non-modifiable factors. Understanding such factors allow healthcare professionals to stratify patients according to their risk of depression or depressive symptoms after hip fracture [44]. For example, the G allele of the 5HTR1A polymorphism, a serotonin receptor, is associated with depressive symptoms 1 year after hip fracture in the older population. Due to the connection between genetics and socio-environmental stressors, this association was not examined in-depth by the authors. Future research focusing on this may aid in establishing whether specific genotypes are predictive of depression or depressive symptoms after hip fracture enabling targeted intervention for individuals with these genotypes. Pre fracture frailty is also associated with depression after hip fracture. Previous literature demonstrates that the presence of either frailty or depression increases the prevalence and incidence of the other [45]. Therefore, stratifying individuals after hip fracture surgery by frailty status allows those most at risk of developing depression to receive specialised management.

Several predictors identified by this review are themselves depressive symptoms namely personal control, hopelessness, negative affect, apathy, and anxiety. Here it was noted that anxiety [15, 35] and negative affect [30] were predictors of more severe depressive episodes as measured by the Montgomery–Åsberg Depression Rating Scale, and apathy [13] was predictive of minor depressive disorders. One study reported a positive association for personal control and no association for hopelessness with depressive symptoms as measured by the Depression, Anxiety and Stress Scale [28]. This is somewhat surprising given both personal control and hopelessness are considered related and symptoms of depression [46, 47]. Indeed, a lack of personal control often leads to feelings of hopelessness and subsequent clinical depression [48, 49]. The surprising result may be due to the poor methodological quality of the study which was at moderate to high risk of bias across five of six QUIPS domains. Alternatively, the findings may suggest certain depressive symptoms may be related to the severity of specific depressive subtypes. For example, the hopelessness theory of depression hypothesizes the negative causal attribution made by individuals in response to adverse life events creates a sense of hopelessness, which can lead to a distinct cognitively mediated subtype of depression, hopelessness depression [47]. It is possible the Depression, Anxiety and Stress Scale may not be sensitive to this subtype.

Previous literature shows that anxiety and apathy often coexist in complex relationships but are distinct entities [50, 51]. In the general population, the coexistence of general anxiety with depressive symptoms is significant, as is the confounding effect the presence of one has on the other [50]. There is also an overlap between the phenomena of apathy and depression or depressive symptoms [51]. However, the extent to which outcome measures for depression or depressive symptoms are sensitive to this has been discussed in the literature [52]. Previous studies have shown measures of these factors are highly correlated with depression in multiple populations [52]. Therefore, it may not be possible to state whether true anxiety and apathy are prognostic factors of depression or depressive symptoms in this review.

### Strengths and limitations

In this review, screening of published and unpublished literature using broad eligibility criteria (including no language restrictions), data extraction, and quality appraisal were completed in duplicate, reducing the risk of bias. We did not search for registered ongoing studies, which may have led to underestimating the extent of prognostic factors. We employed the Quality In Prognosis Studies (QUIPS) tool for the quality assessments. The tool recommends reviewers give an overall risk of bias judgement to studies by deciding the most important domain a priori; the assigned judgement for these specific domains is then used to determine the overall study risk of bias [53]. However, this may potentially lead to bias as the selection of the most important domains is subjective as there is no literature on which domains are the most significant [54]. We, therefore, did not provide a judgement on the overall risk of bias. We were not able to perform quantitative synthesis due to insufficient data; this limited our ability to provide a scientifically rigorous summary of the results [55]. We also adopted broad eligibility criteria and did not set a minimum sample size. This led to the inclusion of studies of varying methodological quality, including those with a small sample size. These studies have poor precision in their estimates due to the small sample, which further limits our ability to draw conclusions from the results.

We employed a broad definition of depression and/or depressive symptoms in our eligibility criteria to identify all potentially relevant literature. However, it is possible factors prognostic of depressive symptoms may/may not be prognostic of a clinical diagnosis of depression. This may have led to an overestimation or an underestimation of the extent of relevant prognostic factors. Additionally, two studies [15, 30] appear to be from the same population (unable to confirm with authors) which may have led to an overestimation of the number of studies reporting no association between age or gender and depression/depressive symptoms after hip fracture.

We did not perform a quantitative synthesis due to insufficient data. This decision was made following review of data extraction. We identified 14 prognostic factors that were reported by more than one study. For each of these factors, no study explicitly identified a primary prognostic factor of interest and associated appropriate potential confounders, rather interpreting multiple effect estimates from one regression model. As previously specified, this approach is not recommended [36] due to risk of bias known as the Table 2 Fallacy [36]. Unfortunately, for seven prognostic factors, the multivariable analysis was not accompanied by univariable analyses for each prognostic factor limiting the potential for meta-analysis from univariable results. Where univariable analysis was reported, there was heterogeneity in factor measurement e.g., length of stay was measured by surgical [13] and by total length of stay [14] or outcome measurement e.g., comorbidities as a prognostic factor of depressive symptoms [31] and of major depressive disorders [15] or in effect estimates e.g., linear regression [33] and logistic regression [29] to evaluate the prognostic association between activities of daily living and depressive symptoms, and insufficient crude data provided to generate new comparable estimates. Therefore, we did not deem the evidence sufficiently homogenous to warrant exploration with quantitative synthesis. This limited our ability to provide a more rigorous summary of the results [55].

This review focused on those with hip fracture, therefore the results may not be generalisable to the older adult population. We excluded studies with a non-hip fracture control group where the study’s results were limited to comparisons between those with and without hip fracture. We took this approach so the results could be directly applied to the hip fracture population however, this may have led to an underestimation of the number of predictors. Further, we did not include data from outcome measures whose sub-components may include questions related to depressive symptoms e.g., EQ-5D. This may have led to an underestimation of the extent to which prognostic factors of depression or depressive symptoms after hip fracture have been explored in the available literature.

## Conclusions

The current review identified 37 prognostic factors of depression or depressive symptoms after hip fracture surgery across 12 studies. Where factors were investigated by more than one study, there was often conflicting evidence and no proposed mechanism for the reported associations. It is therefore not possible to make any clinical recommendations based on the available evidence. Further high-quality research investigating prognostic factors is warranted to inform future intervention and/or stratified approaches to care after hip fracture.

## Supplementary Information

**Additional file 1: Appendix 1.** Search strategies.

**Additional file 2.**

## Data Availability

Data sharing does not apply to this article as no datasets were generated or analysed during the current study.

## Abbreviations

PRISMA: Preferred Reporting Items for Systematic reviews and Meta-Analyses
QUIPS: QUality In Prognosis Studies
ADLs: Activities of Daily Living
