# Supplementary material for: Prognostic factors of depression and depressive symptoms after hip fracture surgery: systematic review
Source: BMC Geriatr. 2021 Oct 10;21:537. doi: 10.1186/s12877-021-02514-1 (PMC8502369; doi:10.1186/s12877-021-02514-1)
Supplement: Supplementary file 1 — Additional file 1: Appendix 1. Search strategies. [file 12877_2021_2514_MOESM1_ESM.docx]

**Appendix 1 - Search strategies**

**MEDLINE**

1. exp Hip Fractures/

2. femoral neck fracture*.mp.

3. ((hip* or femur* or femoral* or trochant* or pertrochant* or intertrochant* or subtrochant* or intracapsular* or extracapsular*) adj3 fracture*).mp. [mp=title, abstract, original title, name of substance word, subject heading word, floating sub-heading word, keyword heading word, organism supplementary concept word, protocol supplementary concept word, rare disease supplementary concept word, unique identifier, synonyms]

4. ((femur* or femoral*) adj3 (neck or proximal or head) adj4 fracture*).mp. [mp=title, abstract, original title, name of substance word, subject heading word, floating sub-heading word, keyword heading word, organism supplementary concept word, protocol supplementary concept word, rare disease supplementary concept word, unique identifier, synonyms]

5. (osteoporo* adj2 hip* fracture).mp. [mp=title, abstract, original title, name of substance word, subject heading word, floating sub-heading word, keyword heading word, organism supplementary concept word, protocol supplementary concept word, rare disease supplementary concept word, unique identifier, synonyms]

6. pelvi* fracture*.mp.

7. 1 or 2 or 3 or 4 or 5 or 6

8. Depression.mp. or exp Depression/

9. ((depress* or dysthymi* or mood or affect* or adjustment) adj2 disorder*).mp. [mp=title, abstract, original title, name of substance word, subject heading word, floating sub-heading word, keyword heading word, organism supplementary concept word, protocol supplementary concept word, rare disease supplementary concept word, unique identifier, synonyms]

10. (dysthymi* or "depress* symptom" or "affect* symptom").mp. [mp=title, abstract, original title, name of substance word, subject heading word, floating sub-heading word, keyword heading word, organism supplementary concept word, protocol supplementary concept word, rare disease supplementary concept word, unique identifier, synonyms]

11. 8 or 9 or 10

12. Validat$.mp. or Predict$.ti. or Rule$.mp. or (Predict$ and (Outcome$ or Risk$ or Model$)).mp. or ((History or Variable$ or Criteria or Scor$ or Characteristic$ or Finding$ or Factor$) and (Predict$ or Model$ or Decision$ or Identif$ or Prognos$)).mp. or (Decision$.mp. and ((Model$ or Clinical$).mp. or Logistic Models/)) or (Prognostic and (History or Variable$ or Criteria or Scor$ or Characteristic$ or Finding$ or Factor$ or Model$)).mp. [mp=title, abstract, original title, name of substance word, subject heading word, floating sub-heading word, keyword heading word, organism supplementary concept word, protocol supplementary concept word, rare disease supplementary concept word, unique identifier, synonyms]

13. (stratification or discriminat* or c?statistic or AUC or calibration or indices or algorithm or multivariable).mp. [mp=title, abstract, original title, name of substance word, subject heading word, floating sub-heading word, keyword heading word, organism supplementary concept word, protocol supplementary concept word, rare disease supplementary concept word, unique identifier, synonyms]

14. ROC Curve.mp. or exp ROC Curve/

15. exp Area Under Curve/ or Area under the curve.mp.

16. 12 or 13 or 14 or 15

17. 7 and 11 and 16

**Embase**

1. exp hip fracture/

2. femoral neck fracture*.mp.

3. ((hip* or femur* or femoral* or trochant* or pertrochant* or intertrochant* or subtrochant* or intracapsular* or extracapsular*) adj3 fracture*).mp. [mp=title, abstract, heading word, drug trade name, original title, device manufacturer, drug manufacturer, device trade name, keyword, floating subheading word, candidate term word]

4. ((femur* or femoral*) adj3 (neck or proximal or head) adj4 fracture*).mp. [mp=title, abstract, heading word, drug trade name, original title, device manufacturer, drug manufacturer, device trade name, keyword, floating subheading word, candidate term word]

5. (osteoporo* adj2 hip* fracture).mp. [mp=title, abstract, heading word, drug trade name, original title, device manufacturer, drug manufacturer, device trade name, keyword, floating subheading word, candidate term word]

6. pelvi* fracture*.mp. [mp=title, abstract, heading word, drug trade name, original title, device manufacturer, drug manufacturer, device trade name, keyword, floating subheading word, candidate term word]

7. 1 or 2 or 3 or 4 or 5 or 6

8. exp depression/ or depression.mp.

9. ((depress* or dysthymi* or mood or affect* or adjustment) adj2 disorder*).mp. [mp=title, abstract, heading word, drug trade name, original title, device manufacturer, drug manufacturer, device trade name, keyword, floating subheading word, candidate term word]

10. (dysthymi* or "depress* symptom" or "affect* symptom").mp. [mp=title, abstract, heading word, drug trade name, original title, device manufacturer, drug manufacturer, device trade name, keyword, floating subheading word, candidate term word]

11. 8 or 9 or 10

12. Validat$.mp. or Predict$.ti. or Rule$.mp. or (Predict$ and (Outcome$ or Risk$ or Model$)).mp. or ((History or Variable$ or Criteria or Scor$ or Characteristic$ or Finding$ or Factor$) and (Predict$ or Model$ or Decision$ or Identif$ or Prognos$)).mp. or (Decision$.mp. and ((Model$ or Clinical$).mp. or Logistic Models/)) or (Prognostic and (History or Variable$ or Criteria or Scor$ or Characteristic$ or Finding$ or Factor$ or Model$)).mp. [mp=title, abstract, heading word, drug trade name, original title, device manufacturer, drug manufacturer, device trade name, keyword, floating subheading word, candidate term word]

13. (stratification or discriminat* or c?statistic or AUC or calibration or indices or algorithm or multivariable).mp. [mp=title, abstract, heading word, drug trade name, original title, device manufacturer, drug manufacturer, device trade name, keyword, floating subheading word, candidate term word]

14. ROC Curve.mp. or exp receiver operating characteristic/

15. Area Under Curve.mp. or exp area under the curve/

16. 12 or 13 or 14 or 15

17. 7 and 11 and 16

**PsychINFO**

1. hip fracture*.mp.

2. femoral neck fracture*.mp.

3. ((hip* or femur* or femoral* or trochant* or pertrochant* or intertrochant* or subtrochant* or intracapsular* or extracapsular*) adj3 fracture*).mp. [mp=title, abstract, heading word, table of contents, key concepts, original title, tests & measures, mesh]

4. ((femur* or femoral*) adj3 (neck or proximal or head) adj4 fracture*).mp. [mp=title, abstract, heading word, table of contents, key concepts, original title, tests & measures, mesh]

5. (osteoporo* adj2 hip* fracture).mp. [mp=title, abstract, heading word, table of contents, key concepts, original title, tests & measures, mesh]

6. pelvi* fracture*.mp.

7. 1 or 2 or 3 or 4 or 5 or 6

8. exp "Depression (Emotion)"/ or exp Major Depression/ or depression.mp. or exp Recurrent Depression/ or exp Late Life Depression/

9. ((depress* or dysthymi* or mood or affect* or adjustment) adj2 disorder*).mp. [mp=title, abstract, heading word, table of contents, key concepts, original title, tests & measures, mesh]

10. (dysthymi* or "depress* symptom" or "affect* symptom").mp. [mp=title, abstract, heading word, table of contents, key concepts, original title, tests & measures, mesh]

11. 8 or 9 or 10

12. Validat$.mp. or Predict$.ti. or Rule$.mp. or (Predict$ and (Outcome$ or Risk$ or Model$)).mp. or ((History or Variable$ or Criteria or Scor$ or Characteristic$ or Finding$ or Factor$) and (Predict$ or Model$ or Decision$ or Identif$ or Prognos$)).mp. or (Decision$.mp. and ((Model$ or Clinical$).mp. or Logistic Models/)) or (Prognostic and (History or Variable$ or Criteria or Scor$ or Characteristic$ or Finding$ or Factor$ or Model$)).mp. [mp=title, abstract, heading word, table of contents, key concepts, original title, tests & measures, mesh]

13. (stratification or discriminat* or c?statistic or AUC or calibration or indices or algorithm or multivariable).mp. [mp=title, abstract, heading word, table of contents, key concepts, original title, tests & measures, mesh]

14. ROC Curve.mp.

15. Area Under Curve.mp.

16. 12 or 13 or 14 or 15

17. 7 and 11 and 16

**CINAHL**

S17 S7 AND S11 AND S16 Expanders - Apply equivalent subjects

Search modes - Boolean/Phrase Interface - EBSCOhost Research Databases

S16 S12 OR S13 OR S14 OR S15 Expanders - Apply equivalent subjects

Search modes - Boolean/Phrase Interface - EBSCOhost Research Databases

S15 (MM "ROC Curve") OR "Area Under Curve" Expanders - Apply equivalent subjects

Search modes - Boolean/Phrase Interface - EBSCOhost Research Databases

S14 (MM "ROC Curve") Expanders - Apply equivalent subjects

Search modes - Boolean/Phrase Interface - EBSCOhost Research Databases

S13 (stratification or discriminat* or c?statistic or AUC or calibration or indices or algorithm or multivariable). Expanders - Apply equivalent subjects

Search modes - Boolean/Phrase Interface - EBSCOhost Research Databases

S12 Validat$.mp. or Predict$.ti. or Rule$.mp. or (Predict$ and (Outcome$ or Risk$ or Model$)).mp. or ((History or Variable$ or Criteria or Scor$ or Characteristic$ or Finding$ or Factor$) and (Predict$ or Model$ or Decision$ or Identif$ or Prognos$)).mp. or (Decision$.mp. and ((Model$ or Clinical$).mp. or Logistic Models/)) or (Prognostic and (History or Variable$ or Criteria or Scor$ or Characteristic$ or Finding$ or Factor$ or Model$)) Expanders - Apply equivalent subjects

Search modes - Boolean/Phrase Interface - EBSCOhost Research Databases

S11 S8 OR S9 OR S10 Expanders - Apply equivalent subjects

Search modes - Boolean/Phrase Interface - EBSCOhost Research Databases

S10 (dysthymi* or "depress* symptom" or "affect* symptom") Expanders - Apply equivalent subjects

Search modes - Boolean/Phrase Interface - EBSCOhost Research Databases

S9 ((depress* or dysthymi* or mood or affect* or adjustment) N2 disorder*) Expanders - Apply equivalent subjects

Search modes - Boolean/Phrase Interface - EBSCOhost Research Databases

S8 (MM "Depression+") Expanders - Apply equivalent subjects

Search modes - Boolean/Phrase Interface - EBSCOhost Research Databases

S7 S1 OR S2 OR S3 OR S4 OR S5 OR S6 Expanders - Apply equivalent subjects

Search modes - Boolean/Phrase Interface - EBSCOhost Research Databases

S6 pelvi* fracture* Expanders - Apply equivalent subjects

Search modes - Boolean/Phrase Interface - EBSCOhost Research Databases

S5 (osteoporo* N2 hip* fracture) Expanders - Apply equivalent subjects

Search modes - Boolean/Phrase Interface - EBSCOhost Research Databases

S4 ((femur* or femoral*) N3 (neck or proximal or head) N4 fracture*) Expanders - Apply equivalent subjects

Search modes - Boolean/Phrase Interface - EBSCOhost Research Databases

S3 . ((hip* or femur* or femoral* or trochant* or pertrochant* or intertrochant* or subtrochant* or intracapsular* or extracapsular*) N3 fracture*) Expanders - Apply equivalent subjects

Search modes - Boolean/Phrase Interface - EBSCOhost Research Databases

S2 (MM "Hip Fractures+") Expanders - Apply equivalent subjects

Search modes - Boolean/Phrase Interface - EBSCOhost Research Databases

S1 (MM "Femoral Fractures+") Expanders - Apply equivalent subjects

Search modes - Boolean/Phrase Interface - EBSCOhost Research Databases

**Web of Science**

# 25

#24 AND #12 AND #7

Indexes=SCI-EXPANDED, SSCI, A&HCI, CPCI-S, CPCI-SSH, ESCI Timespan=All years Edit

# 24

#23 OR #21 OR #20

Indexes=SCI-EXPANDED, SSCI, A&HCI, CPCI-S, CPCI-SSH, ESCI Timespan=All years Edit

# 23 #22 AND #19

Indexes=SCI-EXPANDED, SSCI, A&HCI, CPCI-S, CPCI-SSH, ESCI Timespan=All years Edit

# 22

#18 OR #17 OR #16

Indexes=SCI-EXPANDED, SSCI, A&HCI, CPCI-S, CPCI-SSH, ESCI Timespan=All years Edit

# 21 ALL=(prognostic and (history or variable$ or criteria or scor$ or characteristic$ or finding$ or factor$ or model$) )

Indexes=SCI-EXPANDED, SSCI, A&HCI, CPCI-S, CPCI-SSH, ESCI Timespan=All years Edit

# 20

ALL=(decision$ and ((model$ or clinical$) or logistic models))

Indexes=SCI-EXPANDED, SSCI, A&HCI, CPCI-S, CPCI-SSH, ESCI Timespan=All years Edit

# 19

ALL=(predict$ or model$ or decision$ or identif$ or prognos$)

Indexes=SCI-EXPANDED, SSCI, A&HCI, CPCI-S, CPCI-SSH, ESCI Timespan=All years Edit

# 18 ALL=(history or variable$ or criteria or scor$ or characteristic$ or finding$ or factor$)

Indexes=SCI-EXPANDED, SSCI, A&HCI, CPCI-S, CPCI-SSH, ESCI Timespan=All years Edit

# 17 ALL=(predict$ and (outcome$ or risk$ or model$) )

Indexes=SCI-EXPANDED, SSCI, A&HCI, CPCI-S, CPCI-SSH, ESCI Timespan=All years Edit

# 16

ALL=(validat$ or predict$ or rule$)

Indexes=SCI-EXPANDED, SSCI, A&HCI, CPCI-S, CPCI-SSH, ESCI Timespan=All years Edit

# 15

ALL=Area Under Curve

Indexes=SCI-EXPANDED, SSCI, A&HCI, CPCI-S, CPCI-SSH, ESCI Timespan=All years Edit

# 14

KP=ROC Curve

Indexes=SCI-EXPANDED, SSCI, A&HCI, CPCI-S, CPCI-SSH, ESCI Timespan=All years Edit

# 13

ALL=(stratification or discriminat* or c?statistic or AUC or calibration or indices or algorithm or multivariable)

Indexes=SCI-EXPANDED, SSCI, A&HCI, CPCI-S, CPCI-SSH, ESCI Timespan=All years Edit

# 12

#11 OR #10 OR #9 OR #8

Indexes=SCI-EXPANDED, SSCI, A&HCI, CPCI-S, CPCI-SSH, ESCI Timespan=All years Edit

# 11

ALL=(major* or minor* depress*)

Indexes=SCI-EXPANDED, SSCI, A&HCI, CPCI-S, CPCI-SSH, ESCI Timespan=All years Edit

# 10

ALL=(dysthymi* or "depress* symptom" or "affect* symptom")

Indexes=SCI-EXPANDED, SSCI, A&HCI, CPCI-S, CPCI-SSH, ESCI Timespan=All years Edit

# 9

ALL=((depress* or dysthymi* or mood or affect* or adjustment) AND disorder*)

Indexes=SCI-EXPANDED, SSCI, A&HCI, CPCI-S, CPCI-SSH, ESCI Timespan=All years Edit

# 8

ALL=Depression

Indexes=SCI-EXPANDED, SSCI, A&HCI, CPCI-S, CPCI-SSH, ESCI Timespan=All years Edit

# 7

#6 OR #5 OR #4 OR #3 OR #2 OR #1

Indexes=SCI-EXPANDED, SSCI, A&HCI, CPCI-S, CPCI-SSH, ESCI Timespan=All years Edit

# 6

ALL=pelvi* fracture*

Indexes=SCI-EXPANDED, SSCI, A&HCI, CPCI-S, CPCI-SSH, ESCI Timespan=All years Edit

# 5

ALL=(osteoporo* AND hip* fracture)

Indexes=SCI-EXPANDED, SSCI, A&HCI, CPCI-S, CPCI-SSH, ESCI Timespan=All years Edit

# 4

ALL=((femur* or femoral*) AND (neck or proximal or head) AND fracture*)

Indexes=SCI-EXPANDED, SSCI, A&HCI, CPCI-S, CPCI-SSH, ESCI Timespan=All years Edit

# 3

ALL=((hip* OR femur* OR femoral* OR trochant* OR pertrochant* OR intertrochant* OR subtrochant* OR intracapsular* OR extracapsular*) AND fracture*)

Indexes=SCI-EXPANDED, SSCI, A&HCI, CPCI-S, CPCI-SSH, ESCI Timespan=All years Edit

# 2

ALL="femoral neck fracture*"

Indexes=SCI-EXPANDED, SSCI, A&HCI, CPCI-S, CPCI-SSH, ESCI Timespan=All years Edit

# 1

ALL="hip fracture*"

Indexes=SCI-EXPANDED, SSCI, A&HCI, CPCI-S, CPCI-SSH, ESCI Timespan=All years
