# Supplementary material for: Prognostic factors of depression and depressive symptoms after hip fracture surgery: systematic review
Source: BMC Geriatr. 2021 Oct 10;21:537. doi: 10.1186/s12877-021-02514-1 (PMC8502369; doi:10.1186/s12877-021-02514-1)
Supplement: Supplementary file 2 — Additional file 2. [file 12877_2021_2514_MOESM2_ESM.docx]

| **QUIPS Risk of Bias Assessment Instrument for Prognostic Factor Studies** | | |  |  |
| --- | --- | --- | --- | --- |
| **Author and year of publication** | Lenze, 2008, USA |  |  |  |
| **Study identifier** |  |  |  |  |
| **Reviewers** | RMC and GM |  |  |  |
|  |  |  |  |  |
| **Biases** | **Issues to consider for judging overall rating of "Risk of bias"** | **Study Methods & Comments** | **Rating of reporting** | **Rating of "Risk of bias"** |
| **1. Study Participation** | **Goal: To judge the risk of selection bias (likelihood that relationship between *PF* and *outcome* is different for participants and eligible non-participants).** |  |  |  |
| *Source of target population* | The source population or population of interest is adequately described | Subjects had to be ambulatory prior to the fracture and have a Mini-Mental Status Exam score greater than 20 to participate. Inclusion/Exclusion criteria described | Yes |  |
| *Method used to identify population* | The sampling frame and recruitment are adequately described, including methods to identify the sample sufficient to limit potential bias (number and type used, e.g., referral patterns in health care) | The study recruited females aged 65+ admitted to three Baltimore area hospitals from 1998–2004 who suffered a hip fracture within 72 hours of their hospitalization, had surgical repair of their hip, and had been living in the community prior to the fracture. Sample sufficient to limit potential bias not described | Partial |  |
| *Recruitment period* | Period of recruitment is adequately described | 1998–2004 | Yes |  |
| *Place of recruitment* | Place of recruitment (setting and geographic location) are adequately described | Three Baltimore area hospitals | Yes |  |
| *Inclusion and exclusion criteria* | Inclusion and exclusion criteria are adequately described (e.g., including explicit diagnostic criteria or “zero time” description). | Exclusions included presence of cardiovascular disease, Parkinson’s disease, amyotrophic lateral sclerosis, multiple sclerosis, seizure disorder,  respiratory conditions, diseases of the bone (such as Paget’s disease or osteomalacia), metastatic cancer, cirrhosis, end-stage renal disease, dementia, alcohol abuse, narcotic or benzodiazepine use, or recent gastrointestinal bleeding. Subjects had to be ambulatory prior to the fracture and have a Mini-Mental Status Exam score greater than 20 to participate. | Yes |  |
| *Adequate study participation* | There is adequate participation in the study by eligible individuals | Among the 180 participants enrolled, two were excluded for dementia, three did not provide DNA for genotyping and 30 provided DNA that produced inconclusive results; thus, 145 subjects comprised the sample for this analysis. | Yes |  |
| *Baseline characteristics* | The baseline study sample (i.e., individuals entering the study) is adequately described | Table 1 | Yes |  |
| **Summary Study participation** | **The study sample represents the population of interest on key characteristics, sufficient to limit potential bias of the observed relationship between PF and outcome.** |  |  | Low |
|  |  |  |  |  |
| **2. Study Attrition** | **Goal: To judge the risk of attrition bias (likelihood that relationship between *PF* and *outcome* are different for completing and non-completing participants).** |  |  |  |
| *Proportion of baseline sample available for analysis* | Response rate (i.e., proportion of study sample completing the study and providing outcome data) is adequate. | Data is given for all 145 participants | Yes |  |
| *Attempts to collect information on participants who dropped out* | Attempts to collect information on participants who dropped out of the study are described. | None dropped out; some had missing genotypes | N/A |  |
| *Reasons and potential impact of subjects lost to follow-up* | Reasons for loss to follow-up are provided. | N/A | N/A |  |
| *Outcome and prognostic factor information on those lost to follow-up* | Participants lost to follow-up are adequately described | N/A | N/A |  |
|  | There are no important differences between key characteristics and outcomes in participants who completed the study and those who did not. | The comparison between those with missing and observed genotypes suggested potential selection bias. Those whose genotypes could not be determined were older, had more pre-fracture LPADL, and had more prefracture IADL disability | Yes |  |
| **Study Attrition Summary** | **Loss to follow-up (from baseline sample to study population analysed) is not associated with key characteristics (i.e., the study data adequately represent the sample) sufficient to limit potential bias to the observed relationship between PF and outcome.** |  |  | Moderate |
|  |  |  |  |  |
| **3. Prognostic Factor Measurement** | **Goal: To judge the risk of measurement bias related to how PF was measured (differential measurement of PF related to the level of outcome).** |  |  |  |
| *Definition of the PF* | A clear definition or description of 'PF' is provided (e.g., including dose, level, duration of exposure, and clear specification of the method of measurement). | 5HTR1A is an autoinhibitor of serotonin release and 5HTR2A is a mediator of the downstream effects of serotonin | Partial |  |
| *Valid and Reliable Measurement of PF* | Method of PF measurement is adequately valid and reliable to limit misclassification bias (e.g., may include relevant outside sources of information on measurement properties, also characteristics, such as blind measurement and limited reliance on recall). | Genetic samples were provided from blood draws which were obtained from all subjects within 15 days of hip fracture, which were frozen and later shipped for genetic analysis. High molecular weight DNA was isolated from frozen cell pellets by standard procedures, and the 5HTR1A (−1019) (rs 6925) and 5HTR2AA (−1438) (rs 6311) were genotyped by the fluorescence polarization method of Chen et al (1999). | Yes |  |
|  | Continuous variables are reported or appropriate cut-points (i.e., not data-dependent) are used. | N/A | N/A |  |
| *Method and Setting of PF Measurement* | The method and setting of measurement of PF is the same for all study participants. | Yes, see above | Yes |  |
| *Proportion of data on PF available for analysis* | Adequate proportion of the study sample has complete data for PF variable. | 145 study participants with determined genotypes, ten had no risk alleles, 85 had risk alleles at both genes, 15 had a risk allele at 5HTR1A only, 34 had a risk allele at 5HTR2A only, and one had a risk allele at 5HTR1A but inconclusive results at 5HTR2A | Yes |  |
| *Method used for missing data* | Appropriate methods of imputation are used for missing 'PF' data. | N/A | N/A |  |
| **PF Measurement Summary** | ***PF* is adequately measured in study participants to sufficiently limit potential bias.** |  |  | Low |
|  |  |  |  |  |
| **4. Outcome Measurement** | **Goal: To judge the risk of bias related to the measurement of outcome (differential measurement of outcome related to the baseline level of PF).** |  |  |  |
| *Definition of the Outcome* | A clear definition of outcome is provided, including duration of follow-up and level and extent of the outcome construct. | Several measures assessed clinical status within 15 days post-fracture and then two, six-, and 12-months post-fracture | Partial |  |
| *Valid and Reliable Measurement of Outcome* | The method of outcome measurement used is adequately valid and reliable to limit misclassification bias (e.g., may include relevant outside sources of information on measurement properties, also characteristics, such as blind measurement and confirmation of outcome with valid and reliable test). | The 15-item version of the Geriatric Depression Scale (GDS), a valid method of screening for, and measuring severity of, depressive symptoms in medically ill elderly persons | Yes |  |
| *Method and Setting of Outcome Measurement* | The method and setting of outcome measurement is the same for all study participants. | As above | Yes |  |
| **Outcome Measurement Summary** | ***Outcome of interest* is adequately measured in study participants to sufficiently limit potential bias.** | Specific definition of depressive symptoms not described |  | Moderate |
|  |  |  |  |  |
| **5. Study Confounding** | **Goal: To judge the risk of bias due to confounding (i.e. the effect of PF is distorted by another factor that is related to PF and outcome).** |  |  |  |
| *Important Confounders Measured* | All-important confounders, including treatments are measured. | Time since hip fracture, baseline age [truncated at 90], levels of education, Charlson comorbidity score, pre-fracture LPADLs, and pre-fracture IADLs | Yes |  |
| *Definition of the confounding factor* | Clear definitions of the important confounders measured are provided (e.g., including dose, level, and duration of exposures). | See below | Yes |  |
| *Valid and Reliable Measurement of Confounders* | Measurement of all important confounders is adequately valid and reliable (e.g., may include relevant outside sources of information on measurement properties, also characteristics, such as blind measurement and limited reliance on recall). | A modified Charlson score, an index of comorbid conditions (Charlson et al, 1987); an 11-item Lower extremity Physical Activities of Daily Living (LPADL) scale (items: walking across a room; walking one block; climbing stairs; getting into a car; getting into and out of bed; rising from a chair; putting on pants; putting on socks and shoes; bathtub transfer; taking a bath/shower; and toilet transfer); and a seven-item Instrumental Activities of Daily Living (IADL) scale (items: using telephone; getting to places out of walking distance; shopping for groceries; preparing meals; housecleaning; handling money; taking medications). | Yes |  |
| *Method and Setting of Confounding Measurement* | The method and setting of confounding measurement are the same for all study participants. | Demographics were determined by self-report. Several measures assessed clinical status within 15 days post-fracture and then two, six-, and 12-months post-fracture | Yes |  |
| *Method used for missing data* | Appropriate methods are used if imputation is used for missing confounder data. | Pre- and post-fracture characteristics were examined for different genotypes of 5HTR1A and 5HTR2A, and for those with conclusive genotyping results vs. those with inconclusive results, using two-sample t-tests and Fisher’s exact tests.  The GEE results were robust to assumptions about missingness: most estimates using WEE increased in magnitude compared to the GEE estimates, and no significant associations using GEE became insignificant after using WEE (not shown). | Yes |  |
| *Appropriate Accounting for Confounding* | Important potential confounders are accounted for in the study design (e.g., matching for key variables, stratification, or initial assembly of comparable groups). |  | Unsure |  |
|  | Important potential confounders are accounted for in the analysis (i.e., appropriate adjustment). | Controlling for time since hip fracture, age, comorbid conditions, pre-fracture LPADLs and IADLs, and 5HTR2A | Yes |  |
| **Study Confounding Summary** | **Important potential confounders are appropriately accounted for, limiting potential bias with respect to the relationship between *PF* and *outcome*.** |  |  | Low |
|  |  |  |  |  |
| **6. Statistical Analysis and Reporting** | **Goal: To judge the risk of bias related to the statistical analysis and presentation of results.** |  |  |  |
| *Presentation of analytical strategy* | There is sufficient presentation of data to assess the adequacy of the analysis. | Table 2 | Partial |  |
| *Model development strategy* | The strategy for model building (i.e., inclusion of variables in the statistical model) is appropriate and is based on a conceptual framework or model. | General linear regression models. For each model (5HTR1A and 5HTR2A separately and combined additively) two models were fit: one adjusted for time since hip fracture and the other adjusted for time since fracture and baseline/pre-fracture covariates (baseline age [truncated at 90], levels of education, Charlson comorbidity score, pre-fracture LPADLs, and pre-fracture IADLs). We did not control for ethnicity, as only six participants were African American (rest Caucasian). We controlled for time by including indicator variables for follow-up visits at 2, 6, and 12 months. | Yes |  |
|  | The selected statistical model is adequate for the design of the study. | As above | Yes |  |
| *Reporting of results* | There is no selective reporting of results. | Table 2 | Partial |  |
| **Statistical Analysis and Presentation Summary** | **The statistical analysis is appropriate for the design of the study, limiting potential for presentation of invalid or spurious results.** |  |  | Moderate |
|  |  |  |  |  |
| Modified from: Hayden JA, Côté P, Bombardier C. Evaluation of the Quality of Prognosis Studies in Systematic Reviews. Annals of Internal Medicine. 2006;144:427-437. | | |  |  |

| **QUIPS Risk of Bias Assessment Instrument for Prognostic Factor Studies** | | |  |  |
| --- | --- | --- | --- | --- |
| **Author and year of publication** | Bruggermann et al 2007, Australia |  |  |  |
| **Study identifier** |  |  |  |  |
| **Reviewer** | RMC and GM |  |  |  |
|  |  |  |  |  |
| **Biases** | **Issues to consider for judging overall rating of "Risk of bias"** | **Study Methods & Comments** | **Rating of reporting** | **Rating of "Risk of bias"** |
| Instructions to assess the risk of each potential bias: | These issues will guide your thinking and judgment about the overall risk of bias within each of the 6 domains. Some 'issues' may not be relevant to the specific study or the review research question. These issues are taken together to inform the overall judgment of potential bias for each of the 6 domains. | Provide comments or text exerpts in the white boxes below, as necessary, to facilitate the consensus process that will follow. | Rate the adequacy of reporting as yes, partial, no or unsure. | Rate potential risk of bias for each of the 6 domains as High, Moderate, or Low considering all relevant issues |
| **1. Study Participation** | **Goal: To judge the risk of selection bias (likelihood that relationship between *PF* and *outcome* is different for participants and eligible non-participants).** |  |  |  |
| *Source of target population* | The source population or population of interest is adequately described | A final sample of 103 participants with a mean age of 78.04 (SD = 11.29) who were orthopaedic inpatients admitted to the Flinders Medical Centre  following a hip fracture were recruited, of whom 79% (n = 81) were female. Participants were interviewed on average 5.03 days following surgery | Yes |  |
| *Method used to identify population* | The sampling frame and recruitment are adequately described, including methods to identify the sample sufficient to limit potential bias (number and type used, e.g., referral patterns in health care) | A final sample of 103 participants with a mean age of 78.04 (SD = 11.29) who were orthopaedic inpatients admitted to the Flinders Medical Centre  following a hip fracture were recruited | Partial |  |
| *Recruitment period* | Period of recruitment is adequately described | Not described | No |  |
| *Place of recruitment* | Place of recruitment (setting and geographic location) are adequately described | Inpatients admitted to the Flinders Medical Centre | Partial |  |
| *Inclusion and exclusion criteria* | Inclusion and exclusion criteria are adequately described (e.g., including explicit diagnostic criteria or “zero time” description). | Participants were excluded if hip fracture was non-traumatic (i.e., disease-related), they had dementia, active psychosis, intellectual impairment, moderate-to-severe brain injury, were not proficient in English literacy, and/or were actively suicidal. | Yes |  |
| *Adequate study participation* | There is adequate participation in the study by eligible individuals | Of the 279 patients that were admitted to the hospital with a hip fracture during the study period, 131 were suitable to be approached. Of those excluded, 108 had dementia, 11 had language difficulties, 4 had an intellectual impairment, 4 were unable to communicate (deaf and blind or expressive dysphasia), 10 had suffered a non-trauma related  pathological fracture caused by cancer or osteoporosis, and 11 were judged by nursing staff to be too physically unwell to be approached. Twenty-eight suitable candidates declined to participate. | Yes |  |
| *Baseline characteristics* | The baseline study sample (i.e., individuals entering the study) is adequately described | A final sample of 103 participants with a mean age of 78.04 (SD = 11.29), of whom 79% (n = 81) were female. No Table 1 with baseline characteristics given | Partial |  |
| **Summary Study participation** | **The study sample represents the population of interest on key characteristics, sufficient to limit potential bias of the observed relationship between PF and outcome.** |  |  | Moderate |
|  |  |  |  |  |
| **2. Study Attrition** | **Goal: To judge the risk of attrition bias (likelihood that relationship between *PF* and *outcome* are different for completing and non-completing participants).** |  |  |  |
| *Proportion of baseline sample available for analysis* | Response rate (i.e., proportion of study sample completing the study and providing outcome data) is adequate. | Of the 103 that participated at the Time 1 assessment (T1), 64% (n = 66) took part in the follow-up approximately three weeks later | Yes |  |
| *Attempts to collect information on participants who dropped out* | Attempts to collect information on participants who dropped out of the study are described. | Not described | No |  |
| *Reasons and potential impact of subjects lost to follow-up* | Reasons for loss to follow-up are provided. | Three participants did not complete the T1 assessment due to fatigue or distress. Reasons for not participating at the Time 2 assessment (T2) were: 2 declined to participate in the follow-up at T1, 1 had died, 10 were too cognitively disorientated, 11 stated that they were too unwell and 3 were unable to be contacted. | Partial |  |
| *Outcome and prognostic factor information on those lost to follow-up* | Participants lost to follow-up are adequately described | Participants who did not participate in the T2 assessment had significantly higher hopelessness scores at T1 than those interviewed at T2, t(95) = 2.22, p = .029. The two groups did not differ either in terms of demographics or any other variables. | Partial |  |
|  | There are no important differences between key characteristics and outcomes in participants who completed the study and those who did not. | Participants who did not participate in the T2 assessment had significantly higher hopelessness scores at T1 than those interviewed at T2, t(95) = 2.22, p = .029. The two groups did not differ either in terms of demographics or any other variables. | Yes |  |
| **Study Attrition Summary** | **Loss to follow-up (from baseline sample to study population analyzed) is not associated with key characteristics (i.e., the study data adequately represent the sample) sufficient to limit potential bias to the observed relationship between PF and outcome.** |  |  | High |
|  |  |  |  |  |
| **3. Prognostic Factor Measurement** | **Goal: To judge the risk of measurement bias related to how PF was measured (differential measurement of PF related to the level of outcome).** |  |  |  |
| *Definition of the PF* | A clear definition or description of 'PF' is provided (e.g., including dose, level, duration of exposure, and clear specification of the method of measurement). | Injury beliefs. Consequences and Personal Control – description is unclear | Partial |  |
| *Valid and Reliable Measurement of PF* | Method of PF measurement is adequately valid and reliable to limit misclassification bias (e.g., may include relevant outside sources of information on measurement properties, also characteristics, such as blind measurement and limited reliance on recall). | Revised Illness Perception Questionnaire. Based on previous research findings, the two subscales most associated with negative mood from the measure, Consequences and Personal Control, were used from the nine possible subscales. Each subscale consists of 6 items. A 5-point Likert-type scale response set for the subscales was used ranging from strongly agree to strongly disagree, and mean scores are reported | Yes |  |
|  | Continuous variables are reported or appropriate cut-points (i.e., not data-dependent) are used. | N/A | N/A |  |
| *Method and Setting of PF Measurement* | The method and setting of measurement of PF is the same for all study participants. | Not described | No |  |
| *Proportion of data on PF available for analysis* | Adequate proportion of the study sample has complete data for PF variable. | At Time 1 assessment (T1), 64% (n = 66) took part in the follow-up  approximately three weeks later. 26 didn’t participate in T2 | Yes |  |
| *Method used for missing data* | Appropriate methods of imputation are used for missing 'PF' data. | Not described | No |  |
| **PF Measurement Summary** | ***PF* is adequately measured in study participants to sufficiently limit potential bias.** | How authors treated/analysed participants who didn’t provide complete data is not described |  | High |
|  |  |  |  |  |
| **4. Outcome Measurement** | **Goal: To judge the risk of bias related to the measurement of outcome (differential measurement of outcome related to the baseline level of PF).** |  |  |  |
| *Definition of the Outcome* | A clear definition of outcome is provided, including duration of follow-up and level and extent of the outcome construct. | None given | No |  |
| *Valid and Reliable Measurement of Outcome* | The method of outcome measurement used is adequately valid and reliable to limit misclassification bias (e.g., may include relevant outside sources of information on measurement properties, also characteristics, such as blind measurement and confirmation of outcome with valid and reliable test). | Depression Anxiety Stress Scale - The anxiety and depression scales from the short-form version were used. This 14-item measure consists of seven depression and anxiety statements rated on a 4-point Likert-type scale | Yes |  |
| *Method and Setting of Outcome Measurement* | The method and setting of outcome measurement is the same for all study participants. | Self-report measures of anxiety, depression, injury beliefs and pain were administered verbally by L.S.B. Verbal administration was deemed appropriate as the response sets of each questionnaire changed, which at times made the questionnaires difficult to answer if clarification was not provided. The same measures were administered via telephone 3-weeks after the initial assessment | Yes |  |
| **Outcome Measurement Summary** | ***Outcome of interest* is adequately measured in study participants to sufficiently limit potential bias.** |  |  | Low |
|  |  |  |  |  |
| **5. Study Confounding** | **Goal: To judge the risk of bias due to confounding (i.e. the effect of PF is distorted by another factor that is related to PF and outcome).** |  |  |  |
| *Important Confounders Measured* | All-important confounders, including treatments (key variables in conceptual model), are measured. | After controlling for T1 depression symptoms, and T2 pain ratings and physical mobility. Gender and age were unrelated to anxiety or depression,  thus were not controlled in any analyses. | Yes |  |
| *Definition of the confounding factor* | Clear definitions of the important confounders measured are provided (e.g., including dose, level, and duration of exposures). | Not described | No |  |
| *Valid and Reliable Measurement of Confounders* | Measurement of all important confounders is adequately valid and reliable (e.g., may include relevant outside sources of information on measurement properties, also characteristics, such as blind measurement and limited reliance on recall). | A rating of the average amount of pain the participant had been experiencing was obtained using a 10-point Likert-type scale.  Physical mobility was measured at the second assessment using an adaptation of the instrument devised by Cummings et al. (1988) for hip fracture patients. The 10-item questionnaire asked the participants to indicate their difficulty in performing functional tasks involving weight-bearing on their hip | Yes |  |
| *Method and Setting of Confounding Measurement* | The method and setting of confounding measurement are the same for all study participants. | Self-report measures of anxiety, depression, injury beliefs and pain were administered verbally by L.S.B. Verbal administration was deemed appropriate as the response sets of each questionnaire changed, which at times made the questionnaires difficult to answer if clarification was not provided. The same measures were administered via telephone 3-weeks after the initial assessment, as was a measure of physical mobility. | Yes |  |
| *Method used for missing data* | Appropriate methods are used if imputation is used for missing confounder data. | Not described | No |  |
| *Appropriate Accounting for Confounding* | Important potential confounders are accounted for in the study design (e.g., matching for key variables, stratification, or initial assembly of comparable groups). | Not described |  |  |
|  | Important potential confounders are accounted for in the analysis (i.e., appropriate adjustment). | After controlling for T1 depression symptoms, and T2 pain ratings and physical mobility. Gender and age were unrelated to anxiety or depression,  thus were not controlled in any analyses. | Yes |  |
| **Study Confounding Summary** | **Important potential confounders are appropriately accounted for, limiting potential bias with respect to the relationship between *PF* and *outcome*.** |  |  | Moderate |
|  |  |  |  |  |
| **6. Statistical Analysis and Reporting** | **Goal: To judge the risk of bias related to the statistical analysis and presentation of results.** |  |  |  |
| *Presentation of analytical strategy* | There is sufficient presentation of data to assess the adequacy of the analysis. | Tables III and IV | Yes |  |
| *Model development strategy* | The strategy for model building (i.e., inclusion of variables in the statistical model) is appropriate and is based on a conceptual framework or model. | Data were screened for normality and outliers according to Tabachnick and Fidell (2001). Although several variables were slightly skewed, their transformation did not alter the pattern of any findings hence the original raw data is reported | Yes |  |
|  | The selected statistical model is adequate for the design of the study. | Multiple regression was used to (a) test mediational hypotheses, and (b) to examine which factors contributed to the prediction of anxiety and depressive symptoms at follow-up | Yes |  |
| *Reporting of results* | There is no selective reporting of results. | Tables include all variables described in text | Yes |  |
| **Statistical Analysis and Presentation Summary** | **The statistical analysis is appropriate for the design of the study, limiting potential for presentation of invalid or spurious results.** |  |  | Low |
|  |  |  |  |  |
| Modified from: Hayden JA, Côté P, Bombardier C. Evaluation of the Quality of Prognosis Studies in Systematic Reviews. Annals of Internal Medicine. 2006;144:427-437. | | |  |  |

| **QUIPS Risk of Bias Assessment Instrument for Prognostic Factor Studies** | | |  |  |
| --- | --- | --- | --- | --- |
| **Author and year of publication** | Langer et al 2015, USA |  |  |  |
| **Study identifier** |  |  |  |  |
| **Reviewer** | RMC and GM |  |  |  |
|  |  |  |  |  |
| **Biases** | **Issues to consider for judging overall rating of "Risk of bias"** | **Study Methods & Comments** | **Rating of reporting** | **Rating of "Risk of bias"** |
| Instructions to assess the risk of each potential bias: | These issues will guide your thinking and judgment about the overall risk of bias within each of the 6 domains. Some 'issues' may not be relevant to the specific study or the review research question. These issues are taken together to inform the overall judgment of potential bias for each of the 6 domains. | Provide comments or text exerpts in the white boxes below, as necessary, to facilitate the consensus process that will follow. | Rate the adequacy of reporting as yes, partial, no or unsure. | Rate potential risk of bias for each of the 6 domains as High, Moderate, or Low considering all relevant issues |
| **1. Study Participation** | **Goal: To judge the risk of selection bias (likelihood that relationship between *PF* and *outcome* is different for participants and eligible non-participants).** |  |  |  |
| *Source of target population* | The source population or population of interest is adequately described | A sample of hip fracture participants (n = 500) | Yes |  |
| *Method used to identify population* | The sampling frame and recruitment are adequately described, including methods to identify the sample sufficient to limit potential bias (number and type used, e.g., referral patterns in health care) | Patients were recruited from the orthopaedic departments of eight St. Louis-area hospitals between 2 and 14 days after surgical hip fracture repair | Yes |  |
| *Recruitment period* | Period of recruitment is adequately described | Not described | No |  |
| *Place of recruitment* | Place of recruitment (setting and geographic location) are adequately described | Eight St. Louis-area hospitals | Yes |  |
| *Inclusion and exclusion criteria* | Inclusion and exclusion criteria are adequately described (e.g., including explicit diagnostic criteria or “zero time” description). | Exclusion criteria included language, visual, or hearing barriers to participation, a nonoperable fracture or refracture through prosthesis, metastatic cancer, inability to provide informed consent or cooperate with protocol, the presence of a current major depressive episode at baseline that predated the hip fracture (or baseline evaluation for nonfracture comparisons), presence of dementia or persistent delirium, or the use of depressogenic medications (e.g., high-dose steroids). | Yes |  |
| *Adequate study participation* | There is adequate participation in the study by eligible individuals | Not described | No |  |
| *Baseline characteristics* | The baseline study sample (i.e., individuals entering the study) is adequately described | Participants were predominately women (n = 446, 73.8%) and White (n = 559, 92.5%), with a mean age of 78.40 years (SD = 8.55; range = 60 to 101). Other ethnicities reported were Black (n = 41, 6.8%) and Asian or Pacific Islander (n = 3, 0.5%). Years of education ranged from 2 to 26 (M = 13.40, SD = 2.94). Table 1 | Yes |  |
| **Summary Study participation** | **The study sample represents the population of interest on key characteristics, sufficient to limit potential bias of the observed relationship between PF and outcome.** |  |  | Moderate |
|  |  |  |  |  |
| **2. Study Attrition** | **Goal: To judge the risk of attrition bias (likelihood that relationship between *PF* and *outcome* are different for completing and non-completing participants).** |  |  |  |
| *Proportion of baseline sample available for analysis* | Response rate (i.e., proportion of study sample completing the study and providing outcome data) is adequate. | Not reported | No |  |
| *Attempts to collect information on participants who dropped out* | Attempts to collect information on participants who dropped out of the study are described. | Not reported | No |  |
| *Reasons and potential impact of subjects lost to follow-up* | Reasons for loss to follow-up are provided. | Not reported | No |  |
| *Outcome and prognostic factor information on those lost to follow-up* | Participants lost to follow-up are adequately described. | Not reported | No |  |
|  | There are no important differences between key characteristics and outcomes in participants who completed the study and those who did not. | Not reported | No |  |
| **Study Attrition Summary** | **Loss to follow-up (from baseline sample to study population analyzed) is not associated with key characteristics (i.e., the study data adequately represent the sample) sufficient to limit potential bias to the observed relationship between PF and outcome.** | Number of participants included in analysis and results tables not described |  | High |
|  |  |  |  |  |
| **3. Prognostic Factor Measurement** | **Goal: To judge the risk of measurement bias related to how PF was measured (differential measurement of PF related to the level of outcome).** |  |  |  |
| *Definition of the PF* | A clear definition or description of 'PF' is provided (e.g., including dose, level, duration of exposure, and clear specification of the method of measurement). | Affect is not a unitary construct, but rather is comprised of both positive and negative affect, both of which relate to depression | Partial |  |
| *Valid and Reliable Measurement of PF* | Method of PF measurement is adequately valid and reliable to limit misclassification bias (e.g., may include relevant outside sources of information on measurement properties, also characteristics, such as blind measurement and limited reliance on recall). | The Positive and Negative Affect Schedule (PANAS; Watson, Clark, & Tellegen, 1988) contains two scales employing a 1 (very slightly or not at all) to 5 (extremely) Likert-type scale. An abbreviated version of the measure that assesses positive activated affect and negative activated affect with 5 items each (Mackinnon et al., 1999) was used. | Yes |  |
|  | Continuous variables are reported or appropriate cut-points (i.e., not data-dependent) are used. | N/A | N/A |  |
| *Method and Setting of PF Measurement* | The method and setting of measurement of PF is the same for all study participants. | Participants were assessed at 8 time points. All measures were  administered verbally, with visual aids when measures were given in person. | Yes |  |
| *Proportion of data on PF available for analysis* | Adequate proportion of the study sample has complete data for PF variable. | About 30% of the patients (151 out of 500) had at least some missing  data. | Yes |  |
| *Method used for missing data* | Appropriate methods of imputation are used for missing 'PF' data. | Multiple imputation analyses were conducted in Amelia II to address missing data, primarily from the predictor variables. Multiple imputation was performed on a dataset that included factor scores for the affect variables (e.g., those outputted from longitudinal invariance models), as well as the predictor and outcome variables in their original form. | Yes |  |
| **PF Measurement Summary** | ***PF* is adequately measured in study participants to sufficiently limit potential bias.** |  |  | Low |
|  |  |  |  |  |
| **4. Outcome Measurement** | **Goal: To judge the risk of bias related to the measurement of outcome (differential measurement of outcome related to the baseline level of PF).** |  |  |  |
| *Definition of the Outcome* | A clear definition of outcome is provided, including duration of follow-up and level and extent of the outcome construct. | Not described | No |  |
| *Valid and Reliable Measurement of Outcome* | The method of outcome measurement used is adequately valid and reliable to limit misclassification bias (e.g., may include relevant outside sources of information on measurement properties, also characteristics, such as blind measurement and confirmation of outcome with valid and reliable test). | The Montgomery Asberg Depression Rating Scale is a 10-item measure of severity of depressive symptoms. A 7-day and a 24-hr version were used. Montgomery and Asberg (1979) reported high interrater reliability and sensitivity to change. In the current study, the 7-day version from baseline and Week 52 was used | Yes |  |
| *Method and Setting of Outcome Measurement* | The method and setting of outcome measurement is the same for all study participants. | Participants were assessed at 8 time points. All measures were  administered verbally, with visual aids when measures were given in person. The baseline appointment usually occurred at the hospital 2 to 7 days post-surgery, and the remaining in-person assessments were conducted at varying locations including hospitals, rehabilitation and/or skilled nursing facilities, and patient homes. | Yes |  |
| **Outcome Measurement Summary** | ***Outcome of interest* is adequately measured in study participants to sufficiently limit potential bias.** |  |  | Low |
|  |  |  |  |  |
| **5. Study Confounding** | **Goal: To judge the risk of bias due to confounding (i.e. the effect of PF is distorted by another factor that is related to PF and outcome).** |  |  |  |
| *Important Confounders Measured* | All-important confounders, including treatments (key variables in conceptual model), are measured. | Not described | No |  |
| *Definition of the confounding factor* | Clear definitions of the important confounders measured are provided (e.g., including dose, level, and duration of exposures). | Not described | No |  |
| *Valid and Reliable Measurement of Confounders* | Measurement of all important confounders is adequately valid and reliable (e.g., may include relevant outside sources of information on measurement properties, also characteristics, such as blind measurement and limited reliance on recall). | Not described | No |  |
| *Method and Setting of Confounding Measurement* | The method and setting of confounding measurement are the same for all study participants. | Not described | No |  |
| *Method used for missing data* | Appropriate methods are used if imputation is used for missing confounder data. | Multiple imputation was performed on a dataset that included factor scores for the affect variables (e.g., those outputted from longitudinal invariance models), as well as the predictor and outcome variables in their original form. Does this include confounders? | Unsure |  |
| *Appropriate Accounting for Confounding* | Important potential confounders are accounted for in the study design (e.g., matching for key variables, stratification, or initial assembly of comparable groups). | Not described | No |  |
|  | Important potential confounders are accounted for in the analysis (i.e., appropriate adjustment). | Not described | No |  |
| **Study Confounding Summary** | **Important potential confounders are appropriately accounted for, limiting potential bias with respect to the relationship between *PF* and *outcome*.** |  |  | High |
|  |  |  |  |  |
| **6. Statistical Analysis and Reporting** | **Goal: To judge the risk of bias related to the statistical analysis and presentation of results.** |  |  |  |
| *Presentation of analytical strategy* | There is sufficient presentation of data to assess the adequacy of the analysis. | Table 3 | Partial |  |
| *Model development strategy* | The strategy for model building (i.e., inclusion of variables in the statistical model) is appropriate and is based on a conceptual framework or model. | Exactly which statistical model used for predictors is unclear | Unsure |  |
|  | The selected statistical model is adequate for the design of the study. | Exactly which statistical model used for predictors is unclear | Unsure |  |
| *Reporting of results* | There is no selective reporting of results. | No – Table 3 | Yes |  |
| **Statistical Analysis and Presentation Summary** | **The statistical analysis is appropriate for the design of the study, limiting potential for presentation of invalid or spurious results.** |  |  | High |
|  |  |  |  |  |
| Modified from: Hayden JA, Côté P, Bombardier C. Evaluation of the Quality of Prognosis Studies in Systematic Reviews. Annals of Internal Medicine. 2006;144:427-437. | | |  |  |

| **QUIPS Risk of Bias Assessment Instrument for Prognostic Factor Studies** | | |  |  |
| --- | --- | --- | --- | --- |
| **Author and year of publication** | Cristancho et al 2016, USA |  |  |  |
| **Study identifier** |  |  |  |  |
| **Reviewer** | RMC and GM |  |  |  |
|  |  |  |  |  |
| **Biases** | **Issues to consider for judging overall rating of "Risk of bias"** | **Study Methods & Comments** | **Rating of reporting** | **Rating of "Risk of bias"** |
| Instructions to assess the risk of each potential bias: | These issues will guide your thinking and judgment about the overall risk of bias within each of the 6 domains. Some 'issues' may not be relevant to the specific study or the review research question. These issues are taken together to inform the overall judgment of potential bias for each of the 6 domains. | Provide comments or text exerpts in the white boxes below, as necessary, to facilitate the consensus process that will follow. | Rate the adequacy of reporting as yes, partial, no or unsure. | Rate potential risk of bias for each of the 6 domains as High, Moderate, or Low considering all relevant issues |
| **1. Study Participation** | **Goal: To judge the risk of selection bias (likelihood that relationship between *PF* and *outcome* is different for participants and eligible non-participants).** |  |  |  |
| *Source of target population* | The source population or population of interest is adequately described | Participants aged ≥60 years were screened for inclusion | Yes |  |
| *Method used to identify population* | The sampling frame and recruitment are adequately described, including methods to identify the sample sufficient to limit potential bias (number and type used, e.g., referral patterns in health care) | We recruited participants with a primary diagnosis of hip fracture admitted for surgical correction at eight area hospitals in St Louis, MO between 2008 and 2012. | Yes |  |
| *Recruitment period* | Period of recruitment is adequately described | 2008 and 2012 | Yes |  |
| *Place of recruitment* | Place of recruitment (setting and geographic location) are adequately described | Eight area hospitals in St Louis, MO | Yes |  |
| *Inclusion and exclusion criteria* | Inclusion and exclusion criteria are adequately described (e.g., including explicit diagnostic criteria or “zero time” description). | Key exclusion criteria were non-ambulatory prior to fracture, current diagnosis of major or minor depressive disorder (i.e. were clinically  depressed at time of fracture), and non-transient moderate to severe cognitive impairment (per chart review and brief bedside cognitive testing). Additional exclusions were metastatic cancer, interferon treatment, inoperable fracture, significant language, visual or hearing impairment, lived more than 1 h away, or inability to consent or cooperate with study  protocol. | Yes |  |
| *Adequate study participation* | There is adequate participation in the study by eligible individuals | Twenty-three participants were not included in the trajectory model due to missing data on the MADRS at baseline and an additional 29 participants were excluded because their probability of membership to one group was <0.70. | Yes |  |
| *Baseline characteristics* | The baseline study sample (i.e., individuals entering the study) is adequately described | Table 1 | Yes |  |
| **Summary Study participation** | **The study sample represents the population of interest on key characteristics, sufficient to limit potential bias of the observed relationship between PF and outcome.** |  |  | Low |
|  |  |  |  |  |
| **2. Study Attrition** | **Goal: To judge the risk of attrition bias (likelihood that relationship between *PF* and *outcome* are different for completing and non-completing participants).** |  |  |  |
| *Proportion of baseline sample available for analysis* | Response rate (i.e., proportion of study sample completing the study and providing outcome data) is adequate. | Twenty-three participants were not included in the trajectory model due to missing data on the MADRS at baseline and an additional 29 participants were excluded because their probability of membership to one group was <0.70. | Yes |  |
| *Attempts to collect information on participants who dropped out* | Attempts to collect information on participants who dropped out of the study are described. | Not described | No |  |
| *Reasons and potential impact of subjects lost to follow-up* | Reasons for loss to follow-up are provided. | As above | Yes |  |
| *Outcome and prognostic factor information on those lost to follow-up* | Participants lost to follow-up are adequately described | Not described | No |  |
|  | There are no important differences between key characteristics and outcomes in participants who completed the study and those who did not. | Not described | No |  |
| **Study Attrition Summary** | **Loss to follow-up (from baseline sample to study population analyzed) is not associated with key characteristics (i.e., the study data adequately represent the sample) sufficient to limit potential bias to the observed relationship between PF and outcome.** |  |  | High |
|  |  |  |  |  |
| **3. Prognostic Factor Measurement** | **Goal: To judge the risk of measurement bias related to how PF was measured (differential measurement of PF related to the level of outcome).** |  |  |  |
| *Definition of the PF* | A clear definition or description of 'PF' is provided (e.g., including dose, level, duration of exposure, and clear specification of the method of measurement). | Independent variables included in the final model were age, gender, CIRS-G, antidepressant use, smoking history, pain ratings, SBT cognitive status, FRS mobility scores, GALES stress ratings, DSSI subscales, anxiety symptoms, history of minor/major depression, and implant type. To determine the most relevant correlates of depressive symptomology after hip fracture, we examined covariates that have been shown in previous studies to contribute to depressive symptoms in older adults  No clear primary PF given | No |  |
| *Valid and Reliable Measurement of PF* | Method of PF measurement is adequately valid and reliable to limit misclassification bias (e.g., may include relevant outside sources of information on measurement properties, also characteristics, such as blind measurement and limited reliance on recall). | Basic activities of daily living (BADLs), instrumental activities of  daily living (IADLs), and mobility were assessed with the Functional Recovery Score (FRS) from the Hospital for Joint Diseases Geriatric Hip Fracture Research Group. At all-time points, participants used a numerical rating scale with a score of 0 indicating no pain and 10 the worst pain. Stressful life events experienced during the year prior to fracture were  assessed with the Geriatric Adverse Life Events Scale. The Duke Social Support Index (DSSI; Landerman et al. 1989) was administered at the  initial visit to evaluate four different dimensions of social support. Anxiety was measured by summing three items (tense, worried, relaxed) selected from the brief version of the State-Trait Anxiety Inventory – State. The Short-Blessed Test (SBT) evaluated baseline cognitive status. The Cumulative Illness Rating Scale for Geriatrics (CIRS-G) evaluated medical  illness burden. | Yes |  |
|  | Continuous variables are reported or appropriate cut-points (i.e., not data-dependent) are used. | Yes – measures section for relevant variables | Yes |  |
| *Method and Setting of PF Measurement* | The method and setting of measurement of PF is the same for all study participants. | Baseline, week 4, and week 52 assessments were conducted in person while assessments at weeks 1, 2, 8, 12, and 26 were performed over the  phone. Trained study personnel performed all assessments. | Yes |  |
| *Proportion of data on PF available for analysis* | Adequate proportion of the study sample has complete data for PF variable. | Table 2. Stated n=305 at bottom, is this for PF? Unclear | Not sure |  |
| *Method used for missing data* | Appropriate methods of imputation are used for missing 'PF' data. | Not described | No |  |
| **PF Measurement Summary** | ***PF* is adequately measured in study participants to sufficiently limit potential bias.** |  |  | Moderate |
|  |  |  |  |  |
| **4. Outcome Measurement** | **Goal: To judge the risk of bias related to the measurement of outcome (differential measurement of outcome related to the baseline level of PF).** |  |  |  |
| *Definition of the Outcome* | A clear definition of outcome is provided, including duration of follow-up and level and extent of the outcome construct. | Not described | No |  |
| *Valid and Reliable Measurement of Outcome* | The method of outcome measurement used is adequately valid and reliable to limit misclassification bias (e.g., may include relevant outside sources of information on measurement properties, also characteristics, such as blind measurement and confirmation of outcome with valid and reliable test). | The Montgomery–Asberg Depression Rating Scale was the primary depression measure. Initial MADRS scores assessed depressive symptoms pre-fracture, as hospitalized patients described their mood during the week prior to fracture. The Structured Clinical Interview for DSM-IV disorders (SCID-IV; First et al. 1996) diagnosed major and minor depressive disorder date of onset. The SCID was administered at the initial visit to assess depressive disorder at time of fracture and lifetime history of depressive disorder. | Yes |  |
| *Method and Setting of Outcome Measurement* | The method and setting of outcome measurement is the same for all study participants. | Baseline, week 4, and week 52 assessments were conducted in person while assessments at weeks 1, 2, 8, 12, and 26 were performed over the | Yes |  |
| **Outcome Measurement Summary** | ***Outcome of interest* is adequately measured in study participants to sufficiently limit potential bias.** |  |  | Low |
|  |  |  |  |  |
| **5. Study Confounding** | **Goal: To judge the risk of bias due to confounding (i.e. the effect of PF is distorted by another factor that is related to PF and outcome).** |  |  |  |
| *Important Confounders Measured* | All-important confounders, including treatments (key variables in conceptual model), are measured. | Independent variables included in the final model were age, gender, CIRS-G, antidepressant use, smoking history, pain ratings, SBT cognitive status, FRS mobility scores, GALES stress ratings, DSSI subscales, anxiety symptoms, history of minor/major depression, and implant type. PF also confounders | Partial |  |
| *Definition of the confounding factor* | Clear definitions of the important confounders measured are provided (e.g., including dose, level, and duration of exposures). | See above; PF section | Partial |  |
| *Valid and Reliable Measurement of Confounders* | Measurement of all important confounders is adequately valid and reliable (e.g., may include relevant outside sources of information on measurement properties, also characteristics, such as blind measurement and limited reliance on recall). | See above; PF section | Yes |  |
| *Method and Setting of Confounding Measurement* | The method and setting of confounding measurement are the same for all study participants. | Baseline, week 4, and week 52 assessments were conducted in person while assessments at weeks 1, 2, 8, 12, and 26 were performed over the | Yes |  |
| *Method used for missing data* | Appropriate methods are used if imputation is used for missing confounder data. | Not described | No |  |
| *Appropriate Accounting for Confounding* | Important potential confounders are accounted for in the study design (e.g., matching for key variables, stratification, or initial assembly of comparable groups). | Not described | No |  |
|  | Important potential confounders are accounted for in the analysis (i.e., appropriate adjustment). | Not described | No |  |
| **Study Confounding Summary** | **Important potential confounders are appropriately accounted for, limiting potential bias with respect to the relationship between *PF* and *outcome*.** |  |  | High |
|  |  |  |  |  |
| **6. Statistical Analysis and Reporting** | **Goal: To judge the risk of bias related to the statistical analysis and presentation of results.** |  |  |  |
| *Presentation of analytical strategy* | There is sufficient presentation of data to assess the adequacy of the analysis. | Table 2; unclear how many participants data included in analysis | Partial |  |
| *Model development strategy* | The strategy for model building (i.e., inclusion of variables in the statistical model) is appropriate and is based on a conceptual framework or model. | Multinomial logistic model - Inclusion of these variables was based on previous research supporting a variable's importance, ensuring variables were not redundant, improvement in model fit, an interpretable MLN coefficient in terms of sign, size, and significance, and/or a significant independent ANOVA or χ2 test. Continuous variables were cantered to improve interpretation of log odds. | Yes |  |
|  | The selected statistical model is adequate for the design of the study. | See above - Yes | Yes |  |
| *Reporting of results* | There is no selective reporting of results. | Unclear see Table 2 query re total number of n | Not sure |  |
| **Statistical Analysis and Presentation Summary** | **The statistical analysis is appropriate for the design of the study, limiting potential for presentation of invalid or spurious results.** |  |  | Moderate |
|  |  |  |  |  |
| Modified from: Hayden JA, Côté P, Bombardier C. Evaluation of the Quality of Prognosis Studies in Systematic Reviews. Annals of Internal Medicine. 2006;144:427-437. | | |  |  |

| **QUIPS Risk of Bias Assessment Instrument for Prognostic Factor Studies** | | |  |  |
| --- | --- | --- | --- | --- |
| **Author and year of publication** | Lenze, 2007, USA |  |  |  |
| **Study identifier** |  |  |  |  |
| **Reviewer** | RMC and KL |  |  |  |
|  |  |  |  |  |
| **Biases** | **Issues to consider for judging overall rating of "Risk of bias"** | **Study Methods & Comments** | **Rating of reporting** | **Rating of "Risk of bias"** |
| Instructions to assess the risk of each potential bias: | These issues will guide your thinking and judgment about the overall risk of bias within each of the 6 domains. Some 'issues' may not be relevant to the specific study or the review research question. These issues are taken together to inform the overall judgment of potential bias for each of the 6 domains. | Provide comments or text exerpts in the white boxes below, as necessary, to facilitate the consensus process that will follow. | Rate the adequacy of reporting as yes, partial, no or unsure. | Rate potential risk of bias for each of the 6 domains as High, Moderate, or Low considering all relevant issues |
| **1. Study Participation** | **Goal: To judge the risk of selection bias (likelihood that relationship between *PF* and *outcome* is different for participants and eligible non-participants).** |  |  |  |
| *Source of target population* | The source population or population of interest is adequately described | Patients consecutively admitted to an acute care hospital with a primary diagnosis of hip fracture were approached. | Yes |  |
| *Method used to identify population* | The sampling frame and recruitment are adequately described, including methods to identify the sample sufficient to limit potential bias (number and type used, e.g., referral patterns in health care) | Patients consecutively admitted | Partial |  |
| *Recruitment period* | Period of recruitment is adequately described | Between March 2002 and October 2004 | Yes |  |
| *Place of recruitment* | Place of recruitment (setting and geographic location) are adequately described | An acute care hospital; University of Pittsburgh Medical Center  Shadyside, a large urban hospital in Pittsburgh, Pennsylvania. | Yes |  |
| *Inclusion and exclusion criteria* | Inclusion and exclusion criteria are adequately described (e.g., including explicit diagnostic criteria or “zero time” description). | Inclusionary criteria were aged 60 and older, ability to sign informed consent, and freedom from metastatic cancer. | Yes |  |
| *Adequate study participation* | There is adequate participation in the study by eligible individuals | One hundred forty-one subjects aged 60 and older who were hospitalized for hip fracture and were able to give informed consent (i.e., no severe delirium or dementia) were approached. Of these, 12 refused the baseline evaluation, two had severe cognitive impairment precluding further participation, and one had MDD before the onset of the hip fracture. Thus, 126 subjects constituted the study group. | Yes |  |
| *Baseline characteristics* | The baseline study sample (i.e., individuals entering the study) is adequately described | These subjects were predominantly Caucasian and female and remained in the acute care hospital for an average 5.8 days. Table 1 | Yes |  |
| **Summary Study participation** | **The study sample represents the population of interest on key characteristics, sufficient to limit potential bias of the observed relationship between PF and outcome.** |  |  | Low |
|  |  |  |  |  |
| **2. Study Attrition** | **Goal: To judge the risk of attrition bias (likelihood that relationship between *PF* and *outcome* are different for completing and non-completing participants).** |  |  |  |
| *Proportion of baseline sample available for analysis* | Response rate (i.e., proportion of study sample completing the study and providing outcome data) is adequate. | One hundred forty-one subjects aged 60 and older who were hospitalized for hip fracture and were able to give informed consent (i.e., no severe delirium or dementia) were approached. Of these, 12 refused the baseline evaluation, two had severe cognitive impairment precluding further participation, and one had MDD before the onset of the hip fracture. Thus, 126 subjects constituted the study group. | Yes |  |
| *Attempts to collect information on participants who dropped out* | Attempts to collect information on participants who dropped out of the study are described. | Not described | No |  |
| *Reasons and potential impact of subjects lost to follow-up* | Reasons for loss to follow-up are provided. | Not described | No |  |
| *Outcome and prognostic factor information on those lost to follow-up* | Participants lost to follow-up are adequately described | Not described | No |  |
|  | There are no important differences between key characteristics and outcomes in participants who completed the study and those who did not. | Not described | No |  |
| **Study Attrition Summary** | **Loss to follow-up (from baseline sample to study population analyzed) is not associated with key characteristics (i.e., the study data adequately represent the sample) sufficient to limit potential bias to the observed relationship between PF and outcome.** |  |  | Low |
|  |  |  |  |  |
| **3. Prognostic Factor Measurement** | **Goal: To judge the risk of measurement bias related to how PF was measured (differential measurement of PF related to the level of outcome).** |  |  |  |
| *Definition of the PF* | A clear definition or description of 'PF' is provided (e.g., including dose, level, duration of exposure, and clear specification of the method of measurement). | To measure potential risk markers for depression, demographic variables and characteristics of the fracture, surgery, and length of surgical stay were gathered. No clear PF | Yes |  |
| *Valid and Reliable Measurement of PF* | Method of PF measurement is adequately valid and reliable to limit misclassification bias (e.g., may include relevant outside sources of information on measurement properties, also characteristics, such as blind measurement and limited reliance on recall). | Apathy was measured using the clinician version of the Apathy  Evaluation Scale (AES). Functional status was measured prefracture (through structured interview during the surgical stay) and post fracture (at the end of the surgical stay) using the 13 motor items of the Functional Independence Measure (FIM). 9 Cognition was measured at the end of the surgical stay using the Mini-Mental State Examination40 and at 2 weeks after the surgical stay using the Mattis Initiation-Perseveration subscale (Mattis I/P) to measure executive function41 (this measure was added after the study had started, resulting in decreased n), and the Logical Memory Task of the Wechsler Adult Intelligence Scale was used to measure memory.42 Delirium was measured at the end of the surgical stay using the Delirium Rating Scale43 (DRS). Social support was measured using the Social Network Index. Medical comorbidity was measured using the Cumulative Illness Rating Scale for Geriatrics (CIRS-G), | Yes |  |
|  | Continuous variables are reported or appropriate cut-points (i.e., not data-dependent) are used. | Not described | No |  |
| *Method and Setting of PF Measurement* | The method and setting of measurement of PF is the same for all study participants. | To examine predictors and correlates of MDD, subjects who developed MDD were compared with those who did not on all demographic and clinical variables | Yes |  |
| *Proportion of data on PF available for analysis* | Adequate proportion of the study sample has complete data for PF variable. | Table 1; all participants provided data | N/A |  |
| *Method used for missing data* | Appropriate methods of imputation are used for missing 'PF' data. | None missing | N/A |  |
| **PF Measurement Summary** | ***PF* is adequately measured in study participants to sufficiently limit potential bias.** |  |  | Low |
|  |  |  |  |  |
| **4. Outcome Measurement** | **Goal: To judge the risk of bias related to the measurement of outcome (differential measurement of outcome related to the baseline level of PF).** |  |  |  |
| *Definition of the Outcome* | A clear definition of outcome is provided, including duration of follow-up and level and extent of the outcome construct. | Major depressive disorder (MDD) | Partial |  |
| *Valid and Reliable Measurement of Outcome* | The method of outcome measurement used is adequately valid and reliable to limit misclassification bias (e.g., may include relevant outside sources of information on measurement properties, also characteristics, such as blind measurement and confirmation of outcome with valid and reliable test). | The mood module of the Primary Care Evaluation of Mental Disorders to diagnose MDD and the 17-item Hamilton Depression Rating Scale (Ham-D) to examine depressive symptoms. | Yes |  |
| *Method and Setting of Outcome Measurement* | The method and setting of outcome measurement is the same for all study participants. | Subjects were assessed at the end of their hospital stay using the mood module of the Primary Care Evaluation of Mental Disorders to diagnose MDD35 and the 17-item Hamilton Depression Rating Scale (Ham-D) to examine depressive symptoms.36 These measures were repeated 2  weeks after hospital discharge and then every 4 weeks thereafter until 26 weeks after hospital discharge.  For subjects found to be in a major depressive episode, whether the  episode was present at the time of the hip fracture or started after the hip fracture was systematically ascertained using methodology from the Structured Clinical Interview for the Diagnostic and Statistical Manual of Mental Disorders, Fourth Revision, axis I disorders37 (SCID) | Yes |  |
| **Outcome Measurement Summary** | ***Outcome of interest* is adequately measured in study participants to sufficiently limit potential bias.** |  |  | Low |
|  |  |  |  |  |
| **5. Study Confounding** | **Goal: To judge the risk of bias due to confounding (i.e. the effect of PF is distorted by another factor that is related to PF and outcome).** |  |  |  |
| *Important Confounders Measured* | All important confounders, including treatments (key variables in conceptual model), are measured. | The same demographic and baseline clinical variables were evaluated as possible correlates of depressive symptoms over time using repeated-measures models (dependent variable: Ham-D). | Yes |  |
| *Definition of the confounding factor* | Clear definitions of the important confounders measured are provided (e.g., including dose, level, and duration of exposures). | Demographic variables and characteristics of the fracture, surgery, and length of surgical stay were gathered. No clear PF | Yes |  |
| *Valid and Reliable Measurement of Confounders* | Measurement of all important confounders is adequately valid and reliable (e.g., may include relevant outside sources of information on measurement properties, also characteristics, such as blind measurement and limited reliance on recall). | The same demographic and baseline clinical variables were evaluated as possible correlates of depressive symptoms over time using repeated-measures models (dependent variable: Ham-D). Measurement methods given in PF section above | Yes |  |
| *Method and Setting of Confounding Measurement* | The method and setting of confounding measurement are the same for all study participants. | Subjects who developed MDD were compared with those who did not on all demographic and clinical variables  Not all validated measures but some are  -Doesn’t say how e.g. demographics are measured | Partial |  |
| *Method used for missing data* | Appropriate methods are used if imputation is used for missing confounder data. | Not described | No |  |
| *Appropriate Accounting for Confounding* | Important potential confounders are accounted for in the study design (e.g., matching for key variables, stratification, or initial assembly of comparable groups). | Not described | No |  |
|  | Important potential confounders are accounted for in the analysis (i.e., appropriate adjustment). | Not described | No |  |
| **Study Confounding Summary** | **Important potential confounders are appropriately accounted for, limiting potential bias with respect to the relationship between *PF* and *outcome*.** |  |  | Moderate |
|  |  |  |  |  |
| **6. Statistical Analysis and Reporting** | **Goal: To judge the risk of bias related to the statistical analysis and presentation of results.** |  |  |  |
| *Presentation of analytical strategy* | There is sufficient presentation of data to assess the adequacy of the analysis. | Table; unclear if univariate or multivariate results | Partial |  |
| *Model development strategy* | The strategy for model building (i.e., inclusion of variables in the statistical model) is appropriate and is based on a conceptual framework or model. | Logistic regressions examining association of these variables with  simultaneous or subsequent development of MDD were performed. Similarly, to examine predictors and correlates of depressive symptoms, a repeated-measures mixed-effects model with Ham-D score over time as dependent variable was carried out, examining demographic and clinical variables first in a univariate model and then in a multivariate model. | Yes |  |
|  | The selected statistical model is adequate for the design of the study. | See above | Yes |  |
| *Reporting of results* | There is no selective reporting of results. | No |  |  |
| **Statistical Analysis and Presentation Summary** | **The statistical analysis is appropriate for the design of the study, limiting potential for presentation of invalid or spurious results.** |  |  | Moderate |
|  |  |  |  |  |
| Modified from: Hayden JA, Côté P, Bombardier C. Evaluation of the Quality of Prognosis Studies in Systematic Reviews. Annals of Internal Medicine. 2006;144:427-437. | | |  |  |

| **QUIPS Risk of Bias Assessment Instrument for Prognostic Factor Studies** | | |  |  |
| --- | --- | --- | --- | --- |
| **Author and year of publication** | Shyu 2009, Taiwan |  |  |  |
| **Study identifier** |  |  |  |  |
| **Reviewer** | RMC and GM |  |  |  |
|  |  |  |  |  |
| **Biases** | **Issues to consider for judging overall rating of "Risk of bias"** | **Study Methods & Comments** | **Rating of reporting** | **Rating of "Risk of bias"** |
| Instructions to assess the risk of each potential bias: | These issues will guide your thinking and judgment about the overall risk of bias within each of the 6 domains. Some 'issues' may not be relevant to the specific study or the review research question. These issues are taken together to inform the overall judgment of potential bias for each of the 6 domains. | Provide comments or text exerpts in the white boxes below, as necessary, to facilitate the consensus process that will follow. | Rate the adequacy of reporting as yes, partial, no or unsure. | Rate potential risk of bias for each of the 6 domains as High, Moderate, or Low considering all relevant issues |
| **1. Study Participation** | **Goal: To judge the risk of selection bias (likelihood that relationship between *PF* and *outcome* is different for participants and eligible non-participants).** |  |  |  |
| *Source of target population* | The source population or population of interest is adequately described | Not described | No |  |
| *Method used to identify population* | The sampling frame and recruitment are adequately described, including methods to identify the sample sufficient to limit potential bias (number and type used, e.g., referral patterns in health care) | A convenience sample was recruited from the trauma wards of a typical 3000-bed medical centre in Taiwan. The sample size calculation was based on McNemar’s test, which is used to test two proportions for a given variable obtained from the same participants. | Yes |  |
| *Recruitment period* | Period of recruitment is adequately described | 2001–2003 | Yes |  |
| *Place of recruitment* | Place of recruitment (setting and geographic location) are adequately described | 3000-bed medical centre in Taiwan | Yes |  |
| *Inclusion and exclusion criteria* | Inclusion and exclusion criteria are adequately described (e.g., including explicit diagnostic criteria or “zero time” description). | The inclusion criteria were: (i) age 60 years or older, (ii) hospitalized for hip fracture, (iii) surgery for internal fixation or arthroplasty, (iv) no severe cognitive impairment [Chinese Mini-Mental State Examination (CMMSE) score <10, Yip et al. 1992, Shyu & Yip 2001] and (v) able to walk  independently before the fracture | Yes |  |
| *Adequate study participation* | There is adequate participation in the study by eligible individuals | Of 298 patients who met these criteria, 158 agreed to  participate. | Yes |  |
| *Baseline characteristics* | The baseline study sample (i.e., individuals entering the study) is adequately described | Table 1 | Yes |  |
| **Summary Study participation** | **The study sample represents the population of interest on key characteristics, sufficient to limit potential bias of the observed relationship between PF and outcome.** |  |  | Low |
|  |  |  |  |  |
| **2. Study Attrition** | **Goal: To judge the risk of attrition bias (likelihood that relationship between *PF* and *outcome* are different for completing and non-completing participants).** |  |  |  |
| *Proportion of baseline sample available for analysis* | Response rate (i.e., proportion of study sample completing the study and providing outcome data) is adequate. | Of the 158 patients who agreed to participate, only 147 completed the Geriatric Depression Scale short form (GDS, Burke et al. 1991) before discharge. At the end of 12 months, only 118 people remained in the  study. | Yes |  |
| *Attempts to collect information on participants who dropped out* | Attempts to collect information on participants who dropped out of the study are described. | N/A | N/A |  |
| *Reasons and potential impact of subjects lost to follow-up* | Reasons for loss to follow-up are provided. | The reasons for loss to follow-up after discharge were mortality (n = 8, 5.4%) and refusal or inability to complete the GDS (n = 21, 14.3%). | Yes |  |
| *Outcome and prognostic factor information on those lost to follow-up* | Participants lost to follow-up are adequately described | Not described | No |  |
|  | There are no important differences between key characteristics and outcomes in participants who completed the study and those who did not. | Patients who refused to participate at admission (n = 140) and those who agreed to participate (n = 158) were not statistically significantly different in  terms of gender, age, type of surgery, literacy, concomitant diseases, prefracture performance of ADLs and CMMSE scores. | Yes |  |
| **Study Attrition Summary** | **Loss to follow-up (from baseline sample to study population analyzed) is not associated with key characteristics (i.e., the study data adequately represent the sample) sufficient to limit potential bias to the observed relationship between PF and outcome.** |  |  | Low |
|  |  |  |  |  |
| **3. Prognostic Factor Measurement** | **Goal: To judge the risk of measurement bias related to how PF was measured (differential measurement of PF related to the level of outcome).** |  |  |  |
| *Definition of the PF* | A clear definition or description of 'PF' is provided (e.g., including dose, level, duration of exposure, and clear specification of the method of measurement). | Predictors of changes in depressive symptoms were gender, age, concomitant illnesses, prefracture performance of ADLs, education (literate or illiterate), emotional-social support and cognitive status. | Yes |  |
| *Valid and Reliable Measurement of PF* | Method of PF measurement is adequately valid and reliable to limit misclassification bias (e.g., may include relevant outside sources of information on measurement properties, also characteristics, such as blind measurement and limited reliance on recall). | The number of concomitant illnesses (e.g. cancer, stroke, heart, renal or liver disease, diabetes mellitus, osteoporosis or dementia) was collected from medical records. Prefracture performance of ADLs was measured by  participants’ self-report on the Chinese Barthel Index. Cognitive function was measured by the CMMSE. Emotional-social support was measured by 11 items from the Medical Outcome Study (MOS) social support survey | Yes |  |
|  | Continuous variables are reported or appropriate cut-points (i.e., not data-dependent) are used. | Predictor variables section | Yes |  |
| *Method and Setting of PF Measurement* | The method and setting of measurement of PF is the same for all study participants. | Prior to discharge, data were also collected on demographic variables and cognitive status (CMMSE); at the 1st month after discharge, data were also collected on emotional support. | Yes |  |
| *Proportion of data on PF available for analysis* | Adequate proportion of the study sample has complete data for PF variable. | Not described | No |  |
| *Method used for missing data* | Appropriate methods of imputation are used for missing 'PF' data. | Not described | No |  |
| **PF Measurement Summary** | ***PF* is adequately measured in study participants to sufficiently limit potential bias.** |  |  | Moderate |
|  |  |  |  |  |
| **4. Outcome Measurement** | **Goal: To judge the risk of bias related to the measurement of outcome (differential measurement of outcome related to the baseline level of PF).** |  |  |  |
| *Definition of the Outcome* | A clear definition of outcome is provided, including duration of follow-up and level and extent of the outcome construct. | Depressive symptoms | Yes |  |
| *Valid and Reliable Measurement of Outcome* | The method of outcome measurement used is adequately valid and reliable to limit misclassification bias (e.g., may include relevant outside sources of information on measurement properties, also characteristics, such as blind measurement and confirmation of outcome with valid and reliable test). | Depressive symptoms were assessed by the Chinese version of the GDS short form | Yes |  |
| *Method and Setting of Outcome Measurement* | The method and setting of outcome measurement is the same for all study participants. | Data on depressive symptoms were collected by face-to face interviews prior to discharge (in hospital) and at 1, 3, 6 and 12 months after hospital discharge (at participants’ homes). | Yes |  |
| **Outcome Measurement Summary** | ***Outcome of interest* is adequately measured in study participants to sufficiently limit potential bias.** |  |  | Low |
|  |  |  |  |  |
| **5. Study Confounding** | **Goal: To judge the risk of bias due to confounding (i.e. the effect of PF is distorted by another factor that is related to PF and outcome).** |  |  |  |
| *Important Confounders Measured* | All-important confounders, including treatments (key variables in conceptual model), are measured. | After adjusting some covariates in the logistic model, variables not stated | No |  |
| *Definition of the confounding factor* | Clear definitions of the important confounders measured are provided (e.g., including dose, level, and duration of exposures). | Not described | No |  |
| *Valid and Reliable Measurement of Confounders* | Measurement of all important confounders is adequately valid and reliable (e.g., may include relevant outside sources of information on measurement properties, also characteristics, such as blind measurement and limited reliance on recall). | Not described | No |  |
| *Method and Setting of Confounding Measurement* | The method and setting of confounding measurement are the same for all study participants. | Not described | No |  |
| *Method used for missing data* | Appropriate methods are used if imputation is used for missing confounder data. | Not described | No |  |
| *Appropriate Accounting for Confounding* | Important potential confounders are accounted for in the study design (e.g., matching for key variables, stratification, or initial assembly of comparable groups). | Not described | No |  |
|  | Important potential confounders are accounted for in the analysis (i.e., appropriate adjustment). | Not described | No |  |
| **Study Confounding Summary** | **Important potential confounders are appropriately accounted for, limiting potential bias with respect to the relationship between *PF* and *outcome*.** |  |  | High |
|  |  |  |  |  |
| **6. Statistical Analysis and Reporting** | **Goal: To judge the risk of bias related to the statistical analysis and presentation of results.** |  |  |  |
| *Presentation of analytical strategy* | There is sufficient presentation of data to assess the adequacy of the analysis. | No table of results given only in text p-values | No |  |
| *Model development strategy* | The strategy for model building (i.e., inclusion of variables in the statistical model) is appropriate and is based on a conceptual framework or model. | Multiple logistic regression to explore predictors for persistent risk of depressive symptoms (GDS score ‡5 at two or more time points after discharge) among patients at risk before discharge and to examine  predictors of occurrence of risk of depressive symptoms among patients not at risk before discharge | Yes |  |
|  | The selected statistical model is adequate for the design of the study. | Above | Yes |  |
| *Reporting of results* | There is no selective reporting of results. | No results shown | No |  |
| **Statistical Analysis and Presentation Summary** | **The statistical analysis is appropriate for the design of the study, limiting potential for presentation of invalid or spurious results.** |  |  | High |
|  |  |  |  |  |
| Modified from: Hayden JA, Côté P, Bombardier C. Evaluation of the Quality of Prognosis Studies in Systematic Reviews. Annals of Internal Medicine. 2006;144:427-437. | | |  |  |

| **QUIPS Risk of Bias Assessment Instrument for Prognostic Factor Studies** | | |  |  |
| --- | --- | --- | --- | --- |
| **Author and year of publication** | Liu 2018, Taiwan |  |  |  |
| **Study identifier** |  |  |  |  |
| **Reviewer** | RMC and GM |  |  |  |
|  |  |  |  |  |
| **Biases** | **Issues to consider for judging overall rating of "Risk of bias"** | **Study Methods & Comments** | **Rating of reporting** | **Rating of "Risk of bias"** |
| Instructions to assess the risk of each potential bias: | These issues will guide your thinking and judgment about the overall risk of bias within each of the 6 domains. Some 'issues' may not be relevant to the specific study or the review research question. These issues are taken together to inform the overall judgment of potential bias for each of the 6 domains. | Provide comments or text exerpts in the white boxes below, as necessary, to facilitate the consensus process that will follow. | Rate the adequacy of reporting as yes, partial, no or unsure. | Rate potential risk of bias for each of the 6 domains as High, Moderate, or Low considering all relevant issues |
| **1. Study Participation** | **Goal: To judge the risk of selection bias (likelihood that relationship between *PF* and *outcome* is different for participants and eligible non-participants).** |  |  |  |
| *Source of target population* | The source population or population of interest is adequately described | This study was a secondary analysis of longitudinal data from a randomized controlled trial in which hip-fracture patients received one of three care models: usual care, interdisciplinary care, and comprehensive care | Yes |  |
| *Method used to identify population* | The sampling frame and recruitment are adequately described, including methods to identify the sample sufficient to limit potential bias (number and type used, e.g., referral patterns in health care) | Secondary analysis of longitudinal data | Yes |  |
| *Recruitment period* | Period of recruitment is adequately described | September 2005 to July 2010 | Yes |  |
| *Place of recruitment* | Place of recruitment (setting and geographic location) are adequately described | Not described | No |  |
| *Inclusion and exclusion criteria* | Inclusion and exclusion criteria are adequately described (e.g., including explicit diagnostic criteria or “zero time” description). | Patients were included by these criteria: (a) 60 years or older, (b)  admitted to hospital from a home setting for accidental single-side hip fracture, (c) received hip arthroplasty or internal fixation, (d) performed full-range motion against gravity and some or full resistance, (e) prefracture Chinese Barthel Index (CBI) score 70, and (f) lived in northern Taiwan. Patients were included in this secondary analysis if they scored 5 on the GDS-s before discharge or in one of four follow-up assessments. | Yes |  |
| *Adequate study participation* | There is adequate participation in the study by eligible individuals | Figure 1 | Yes |  |
| *Baseline characteristics* | The baseline study sample (i.e., individuals entering the study) is adequately described | Table 3 | Yes |  |
| **Summary Study participation** | **The study sample represents the population of interest on key characteristics, sufficient to limit potential bias of the observed relationship between PF and outcome.** |  |  | Low |
|  |  |  |  |  |
| **2. Study Attrition** | **Goal: To judge the risk of attrition bias (likelihood that relationship between *PF* and *outcome* are different for completing and non-completing participants).** |  |  |  |
| *Proportion of baseline sample available for analysis* | Response rate (i.e., proportion of study sample completing the study and providing outcome data) is adequate. | Figure 1 | Yes |  |
| *Attempts to collect information on participants who dropped out* | Attempts to collect information on participants who dropped out of the study are described. | Not described | No |  |
| *Reasons and potential impact of subjects lost to follow-up* | Reasons for loss to follow-up are provided. | Figure 1 | Yes |  |
| *Outcome and prognostic factor information on those lost to follow-up* | Participants lost to follow-up are adequately described | Not described | No |  |
|  | There are no important differences between key characteristics and outcomes in participants who completed the study and those who did not. | These patients and those excluded from this secondary analysis did not differ significantly in demographic or clinical characteristics, except for depressive symptoms. | Yes |  |
| **Study Attrition Summary** | **Loss to follow-up (from baseline sample to study population analyzed) is not associated with key characteristics (i.e., the study data adequately represent the sample) sufficient to limit potential bias to the observed relationship between PF and outcome.** |  |  | Moderate |
|  |  |  |  |  |
| **3. Prognostic Factor Measurement** | **Goal: To judge the risk of measurement bias related to how PF was measured (differential measurement of PF related to the level of outcome).** |  |  |  |
| *Definition of the PF* | A clear definition or description of 'PF' is provided (e.g., including dose, level, duration of exposure, and clear specification of the method of measurement). | Patients were assessed for range of motion, self-care ability, nutritional status, pain intensity, and health-related quality of life. | Yes |  |
| *Valid and Reliable Measurement of PF* | Method of PF measurement is adequately valid and reliable to limit misclassification bias (e.g., may include relevant outside sources of information on measurement properties, also characteristics, such as blind measurement and limited reliance on recall). | Range of motion was assessed as flexion and extension ranges of major joints. Ranges for ankle and hip joints were measured using a universal goniometer. Trunk-forward flexion measured the distance of forward reach while sitting. Trunk-backward extension flexibility measured the difference in distance between the seventh cervical and first spinal processes while sitting and bending backward. Prefracture mobility and selfcare ability was rated at admission by the Chinese Barthel Index. Health-related quality of life was measured using the Taiwan-version SF-36 | Yes |  |
|  | Continuous variables are reported or appropriate cut-points (i.e., not data-dependent) are used. | Raw scores are transformed to values from 0 to 100; higher scores  indicate better health-related quality of life. | Yes |  |
| *Method and Setting of PF Measurement* | The method and setting of measurement of PF is the same for all study participants. | Patients were assessed before discharge and at 1-, 3-, 6-, and 12-months following discharge for depressive symptoms, range of motion, self-care ability, nutritional status, pain intensity, and health-related quality of life. | Yes |  |
| *Proportion of data on PF available for analysis* | Adequate proportion of the study sample has complete data for PF variable. | Not described | No |  |
| *Method used for missing data* | Appropriate methods of imputation are used for missing 'PF' data. | Not described | No |  |
| **PF Measurement Summary** | ***PF* is adequately measured in study participants to sufficiently limit potential bias.** |  |  | Moderate |
|  |  |  |  |  |
| **4. Outcome Measurement** | **Goal: To judge the risk of bias related to the measurement of outcome (differential measurement of outcome related to the baseline level of PF).** |  |  |  |
| *Definition of the Outcome* | A clear definition of outcome is provided, including duration of follow-up and level and extent of the outcome construct. | Depressive symptoms | Yes |  |
| *Valid and Reliable Measurement of Outcome* | The method of outcome measurement used is adequately valid and reliable to limit misclassification bias (e.g., may include relevant outside sources of information on measurement properties, also characteristics, such as blind measurement and confirmation of outcome with valid and reliable test). | Depressive symptoms were assessed by the Chinese-version GDS-s | Yes |  |
| *Method and Setting of Outcome Measurement* | The method and setting of outcome measurement is the same for all study participants. | Patients were assessed before discharge and at 1-, 3-, 6-, and 12-months following discharge for depressive symptoms | Yes |  |
| **Outcome Measurement Summary** | ***Outcome of interest* is adequately measured in study participants to sufficiently limit potential bias.** |  |  | Low |
|  |  |  |  |  |
| **5. Study Confounding** | **Goal: To judge the risk of bias due to confounding (i.e. the effect of PF is distorted by another factor that is related to PF and outcome).** |  |  |  |
| *Important Confounders Measured* | All-important confounders, including treatments (key variables in conceptual model), are measured. | Age, gender, marital status, educational level, comorbidities, cognitive impairment, functional impairment and care model) and group membership probability (i.e., the probability of belonging to a depressive-trajectory group) | Yes |  |
| *Definition of the confounding factor* | Clear definitions of the important confounders measured are provided (e.g., including dose, level, and duration of exposures). | As above | Yes |  |
| *Valid and Reliable Measurement of Confounders* | Measurement of all important confounders is adequately valid and reliable (e.g., may include relevant outside sources of information on measurement properties, also characteristics, such as blind measurement and limited reliance on recall). | Not described | No |  |
| *Method and Setting of Confounding Measurement* | The method and setting of confounding measurement are the same for all study participants. | All baseline characteristics | Yes |  |
| *Method used for missing data* | Appropriate methods are used if imputation is used for missing confounder data. | Not described | No |  |
| *Appropriate Accounting for Confounding* | Important potential confounders are accounted for in the study design (e.g., matching for key variables, stratification, or initial assembly of comparable groups). | Not described | No |  |
|  | Important potential confounders are accounted for in the analysis (i.e., appropriate adjustment). | Not described | No |  |
| **Study Confounding Summary** | **Important potential confounders are appropriately accounted for, limiting potential bias with respect to the relationship between *PF* and *outcome*.** |  |  | Low |
|  |  |  |  |  |
| **6. Statistical Analysis and Reporting** | **Goal: To judge the risk of bias related to the statistical analysis and presentation of results.** |  |  |  |
| *Presentation of analytical strategy* | There is sufficient presentation of data to assess the adequacy of the analysis. | Table 2; full results of each group not reported | Partial |  |
| *Model development strategy* | The strategy for model building (i.e., inclusion of variables in the statistical model) is appropriate and is based on a conceptual framework or model. | Binary logistic regression modelling, with trajectory group as a dependent variable. Models with and without predictors had similar results in terms of model selection. | Yes |  |
|  | The selected statistical model is adequate for the design of the study. | As above | Yes |  |
| *Reporting of results* | There is no selective reporting of results. | Unclear | Unclear |  |
| **Statistical Analysis and Presentation Summary** | **The statistical analysis is appropriate for the design of the study, limiting potential for presentation of invalid or spurious results.** |  |  | High |
|  |  |  |  |  |
| Modified from: Hayden JA, Côté P, Bombardier C. Evaluation of the Quality of Prognosis Studies in Systematic Reviews. Annals of Internal Medicine. 2006;144:427-437. | | |  |  |

| **QUIPS Risk of Bias Assessment Instrument for Prognostic Factor Studies** | | |  |  |
| --- | --- | --- | --- | --- |
| **Author and year of publication** | Matheny et al 2011 |  |  |  |
| **Study identifier** |  |  |  |  |
| **Reviewer** | RMC and GM |  |  |  |
|  |  |  |  |  |
| **Biases** | **Issues to consider for judging overall rating of "Risk of bias"** | **Study Methods & Comments** | **Rating of reporting** | **Rating of "Risk of bias"** |
| Instructions to assess the risk of each potential bias: | These issues will guide your thinking and judgment about the overall risk of bias within each of the 6 domains. Some 'issues' may not be relevant to the specific study or the review research question. These issues are taken together to inform the overall judgment of potential bias for each of the 6 domains. | Provide comments or text exerpts in the white boxes below, as necessary, to facilitate the consensus process that will follow. | Rate the adequacy of reporting as yes, partial, no or unsure. | Rate potential risk of bias for each of the 6 domains as High, Moderate, or Low considering all relevant issues |
| **1. Study Participation** | **Goal: To judge the risk of selection bias (likelihood that relationship between *PF* and *outcome* is different for participants and eligible non-participants).** |  |  |  |
| *Source of target population* | The source population or population of interest is adequately described | BHS-4 enrolled 180 community-dwelling female hip fracture patients 65  and older who had a non-pathologic fracture within 72 hours of admission and surgical repair of the fracture | Yes |  |
| *Method used to identify population* | The sampling frame and recruitment are adequately described, including methods to identify the sample sufficient to limit potential bias (number and type used, e.g., referral patterns in health care) | Patients were drawn from the fourth cohort in the Baltimore Hip Studies (BHS-4), a randomized clinical trial which tested the feasibility of the Exercise Plus Program compared to usual care in hip fracture patients. Participants were enrolled within 15 days of the hip fracture | Yes |  |
| *Recruitment period* | Period of recruitment is adequately described | November 1998 to September 2004 | Yes |  |
| *Place of recruitment* | Place of recruitment (setting and geographic location) are adequately described | Recruitment was initiated in 3 hospitals in the Baltimore area | Yes |  |
| *Inclusion and exclusion criteria* | Inclusion and exclusion criteria are adequately described (e.g., including explicit diagnostic criteria or “zero time” description). | Eligibility was determined through a medical chart review, medical assessment, and cognitive screen. BHS-4 enrolled 180 community-dwelling female hip fracture patients 65 and older who had a non-pathologic fracture within 72 hours of admission and surgical repair of the fracture. Additional eligibility criteria included ability to walk without human  assistance prior to the fracture, no medical conditions contraindicated with exercise, and a score of ≥20 on the Mini-Mental State Examination (MMSE) (28). | Yes |  |
| *Adequate study participation* | There is adequate participation in the study by eligible individuals | Due to safety concerns of older adults exercising independently in the home, the stringent set of study inclusion criteria resulted in only 243 of the 1,276 (19%) screened hip fracture patients being eligible; however, 74% of eligible women enrolled in the trial. The final sample for this analysis  consisted of the 134 unique BHS-4 participants | Yes |  |
| *Baseline characteristics* | The baseline study sample (i.e., individuals entering the study) is adequately described | Table 1 | Yes |  |
| **Summary Study participation** | **The study sample represents the population of interest on key characteristics, sufficient to limit potential bias of the observed relationship between PF and outcome.** |  |  | Low |
|  |  |  |  |  |
| **2. Study Attrition** | **Goal: To judge the risk of attrition bias (likelihood that relationship between *PF* and *outcome* are different for completing and non-completing participants).** |  |  |  |
| *Proportion of baseline sample available for analysis* | Response rate (i.e., proportion of study sample completing the study and providing outcome data) is adequate. | Not described | No |  |
| *Attempts to collect information on participants who dropped out* | Attempts to collect information on participants who dropped out of the study are described. | Not described | No |  |
| *Reasons and potential impact of subjects lost to follow-up* | Reasons for loss to follow-up are provided. | Not described | No |  |
| *Outcome and prognostic factor information on those lost to follow-up* | Participants lost to follow-up are adequately described | Not described | No |  |
|  | There are no important differences between key characteristics and outcomes in participants who completed the study and those who did not. | Not described | No |  |
| **Study Attrition Summary** | **Loss to follow-up (from baseline sample to study population analyzed) is not associated with key characteristics (i.e., the study data adequately represent the sample) sufficient to limit potential bias to the observed relationship between PF and outcome.** |  |  | High |
|  |  |  |  |  |
| **3. Prognostic Factor Measurement** | **Goal: To judge the risk of measurement bias related to how PF was measured (differential measurement of PF related to the level of outcome).** |  |  |  |
| *Definition of the PF* | A clear definition or description of 'PF' is provided (e.g., including dose, level, duration of exposure, and clear specification of the method of measurement). | Age, gender, BMI, cognitive status, lower extremity function | Yes |  |
| *Valid and Reliable Measurement of PF* | Method of PF measurement is adequately valid and reliable to limit misclassification bias (e.g., may include relevant outside sources of information on measurement properties, also characteristics, such as blind measurement and limited reliance on recall). | The medical record was reviewed to obtain the patient’s age and the number of medical comorbidities. Height and weight were used to calculate body mass index. Cognitive status was assessed at baseline using the  Mini-Mental State Examination, Lower Extremity Gain Scale (LEGS) score | Yes |  |
|  | Continuous variables are reported or appropriate cut-points (i.e., not data-dependent) are used. | Not described | No |  |
| *Method and Setting of PF Measurement* | The method and setting of measurement of PF is the same for all study participants. | Not described | No |  |
| *Proportion of data on PF available for analysis* | Adequate proportion of the study sample has complete data for PF variable. | Not described | No |  |
| *Method used for missing data* | Appropriate methods of imputation are used for missing 'PF' data. | The GEE analytic method uses data from all visits with both a  GDS and cytokine measure and enables performing longitudinal analyses when some study visits are missing. | Yes |  |
| **PF Measurement Summary** | ***PF* is adequately measured in study participants to sufficiently limit potential bias.** |  |  | Moderate |
|  |  |  |  |  |
| **4. Outcome Measurement** | **Goal: To judge the risk of bias related to the measurement of outcome (differential measurement of outcome related to the baseline level of PF).** |  |  |  |
| *Definition of the Outcome* | A clear definition of outcome is provided, including duration of follow-up and level and extent of the outcome construct. | Depressive symptoms | Yes |  |
| *Valid and Reliable Measurement of Outcome* | The method of outcome measurement used is adequately valid and reliable to limit misclassification bias (e.g., may include relevant outside sources of information on measurement properties, also characteristics, such as blind measurement and confirmation of outcome with valid and reliable test). | 15-item Geriatric Depression Scale (GDS) | Yes |  |
| *Method and Setting of Outcome Measurement* | The method and setting of outcome measurement is the same for all study participants. | Two, 6- and 12-month depressive symptoms were measured | Yes |  |
| **Outcome Measurement Summary** | ***Outcome of interest* is adequately measured in study participants to sufficiently limit potential bias.** |  |  | Low |
|  |  |  |  |  |
| **5. Study Confounding** | **Goal: To judge the risk of bias due to confounding (i.e. the effect of PF is distorted by another factor that is related to PF and outcome).** |  |  |  |
| *Important Confounders Measured* | All-important confounders, including treatments (key variables in conceptual model), are measured. | The models were adjusted for age, Charlson Comorbidity Index score, MMSE score, treatment group (exercise versus control) | Yes |  |
| *Definition of the confounding factor* | Clear definitions of the important confounders measured are provided (e.g., including dose, level, and duration of exposures). | As above, PF Section | Yes |  |
| *Valid and Reliable Measurement of Confounders* | Measurement of all important confounders is adequately valid and reliable (e.g., may include relevant outside sources of information on measurement properties, also characteristics, such as blind measurement and limited reliance on recall). | As above, PF Section | Yes |  |
| *Method and Setting of Confounding Measurement* | The method and setting of confounding measurement are the same for all study participants. | As above, PF Section | Yes |  |
| *Method used for missing data* | Appropriate methods are used if imputation is used for missing confounder data. | The GEE analytic method uses data from all visits with both a  GDS and cytokine measure and enables performing longitudinal analyses when some study visits are missing. | Yes |  |
| *Appropriate Accounting for Confounding* | Important potential confounders are accounted for in the study design (e.g., matching for key variables, stratification, or initial assembly of comparable groups). | Not described | No |  |
|  | Important potential confounders are accounted for in the analysis (i.e., appropriate adjustment). | The models were adjusted for age, Charlson Comorbidity Index score, MMSE score, treatment group (exercise versus control) | Yes |  |
| **Study Confounding Summary** | **Important potential confounders are appropriately accounted for, limiting potential bias with respect to the relationship between *PF* and *outcome*.** |  |  | Low |
|  |  |  |  |  |
| **6. Statistical Analysis and Reporting** | **Goal: To judge the risk of bias related to the statistical analysis and presentation of results.** |  |  |  |
| *Presentation of analytical strategy* | There is sufficient presentation of data to assess the adequacy of the analysis. | No table of results given, effect estimates and results not given in text apart from some p-values and 95% CI | No |  |
| *Model development strategy* | The strategy for model building (i.e., inclusion of variables in the statistical model) is appropriate and is based on a conceptual framework or model. | Generalized estimating equations (GEE) (35) were used to model the longitudinal relationship between IL-6 and sTNF-αR1category at the 2-, 6- and 12-month follow-up evaluations and GDS scores. In order to examine the role of lower extremity function as a potential mediator of the relationship between inflammation and depressive symptoms additional models adjusted for lower extremity performance as measured by the LEGS score. | Yes |  |
|  | The selected statistical model is adequate for the design of the study. | This method can account for possible correlations in repeated measures  over time and is suitable for exploring differences in values measured at different times. | Yes |  |
| *Reporting of results* | There is no selective reporting of results. | No tables given, summary of results given in text | Partial |  |
| **Statistical Analysis and Presentation Summary** | **The statistical analysis is appropriate for the design of the study, limiting potential for presentation of invalid or spurious results.** |  |  | High |
|  |  |  |  |  |
| Modified from: Hayden JA, Côté P, Bombardier C. Evaluation of the Quality of Prognosis Studies in Systematic Reviews. Annals of Internal Medicine. 2006;144:427-437. | | |  |  |

| **QUIPS Risk of Bias Assessment Instrument for Prognostic Factor Studies** | | |  |  |
| --- | --- | --- | --- | --- |
| **Author and year of publication** | Van de Ree, 2020, the Netherlands |  |  |  |
| **Study identifier** |  |  |  |  |
| **Reviewer** | RMC and KL |  |  |  |
|  |  |  |  |  |
| **Biases** | **Issues to consider for judging overall rating of "Risk of bias"** | **Study Methods & Comments** | **Rating of reporting** | **Rating of "Risk of bias"** |
| Instructions to assess the risk of each potential bias: | These issues will guide your thinking and judgment about the overall risk of bias within each of the 6 domains. Some 'issues' may not be relevant to the specific study or the review research question. These issues are taken together to inform the overall judgment of potential bias for each of the 6 domains. | Provide comments or text exerpts in the white boxes below, as necessary, to facilitate the consensus process that will follow. | Rate the adequacy of reporting as yes, partial, no or unsure. | Rate potential risk of bias for each of the 6 domains as High, Moderate, or Low considering all relevant issues |
| **1. Study Participation** | **Goal: To judge the risk of selection bias (likelihood that relationship between *PF* and *outcome* is different for participants and eligible non-participants).** |  |  |  |
| *Source of target population* | The source population or population of interest is adequately described | Patients with a hip fracture being ≥65 years old were included. | Yes |  |
| *Method used to identify population* | The sampling frame and recruitment are adequately described, including methods to identify the sample sufficient to limit potential bias (number and type used, e.g., referral patterns in health care) | This hip fracture cohort data was derived from the Brabant Injury Outcome Surveillance (BIOS), a multicentre longitudinal prospective cohort study. | Yes |  |
| *Recruitment period* | Period of recruitment is adequately described | All participants were included between August 2015 and November 2016 | Yes |  |
| *Place of recruitment* | Place of recruitment (setting and geographic location) are adequately described | Ten hospitals (Noord Brabant, Netherlands) | Yes |  |
| *Inclusion and exclusion criteria* | Inclusion and exclusion criteria are adequately described (e.g., including explicit diagnostic criteria or “zero time” description). | Patients with a hip fracture being ≥65 years old were included. Exclusion criteria were: (i) patients with cognitive impairment, (ii) pathological hip fractures (iii) and patients with insufficient knowledge of the Dutch language | Yes |  |
| *Adequate study participation* | There is adequate participation in the study by eligible individuals | In total 570 patients were included (69.7% inclusion rate) | Yes |  |
| *Baseline characteristics* | The baseline study sample (i.e., individuals entering the study) is adequately described | Table 1 shows baseline patients’ characteristics. In total, 264 (46.3%) participants were considered as frail and 21 (3.7%) participants had an early-onset dementia, but were capable (with help) to complete the questionnaires. | Yes |  |
| **Summary Study participation** | **The study sample represents the population of interest on key characteristics, sufficient to limit potential bias of the observed relationship between PF and outcome.** |  |  | Low |
|  |  |  |  |  |
| **2. Study Attrition** | **Goal: To judge the risk of attrition bias (likelihood that relationship between *PF* and *outcome* are different for completing and non-completing participants).** |  |  |  |
| *Proportion of baseline sample available for analysis* | Response rate (i.e., proportion of study sample completing the study and providing outcome data) is adequate. | Figure 1; shows the dropouts for each timepoint | Yes |  |
| *Attempts to collect information on participants who dropped out* | Attempts to collect information on participants who dropped out of the study are described. | In case of no return, we endeavored to contact the participant or relative by telephone on several occasions. If this method failed, the participant was considered to be a non-responder at that time point (indicated as ‘no show’ in Fig. 1) | Yes |  |
| *Reasons and potential impact of subjects lost to follow-up* | Reasons for loss to follow-up are provided. | Figure 1; shows the dropouts for each timepoint | Yes |  |
| *Outcome and prognostic factor information on those lost to follow-up* | Participants lost to follow-up are adequately described | Responders of the questionnaires were significantly younger compared to  the non-responders (78.4 [SD: 8.1] and 82.8 [SD: 7.8] years, respectively). Responders were more often healthy (ASA 1 or 2; 70% vs 51%) and had a shorter LOS (mean LOS 8.3 [SD 4.5] vs 9.1 [SD 6.3]) compared to the non-responders. | Partially |  |
|  | There are no important differences between key characteristics and outcomes in participants who completed the study and those who did not. | Responders of the questionnaires were significantly younger compared to  the non-responders (78.4 [SD: 8.1] and 82.8 [SD: 7.8] years, respectively). Responders were more often healthy (ASA 1 or 2; 70% vs 51%) and had a shorter LOS (mean LOS 8.3 [SD 4.5] vs 9.1 [SD 6.3]) compared to the non-responders. | Yes |  |
| **Study Attrition Summary** | **Loss to follow-up (from baseline sample to study population analyzed) is not associated with key characteristics (i.e., the study data adequately represent the sample) sufficient to limit potential bias to the observed relationship between PF and outcome.** |  |  | Moderate |
|  |  |  |  |  |
| **3. Prognostic Factor Measurement** | **Goal: To judge the risk of measurement bias related to how PF was measured (differential measurement of PF related to the level of outcome).** |  |  |  |
| *Definition of the PF* | A clear definition or description of 'PF' is provided (e.g., including dose, level, duration of exposure, and clear specification of the method of measurement). | Age, gender, body mass index (BMI), educational level, prefracture residential status, prefracture mobility, American Society of Anesthesiologists grading (ASA), type of fracture, type of treatment, length of hospital stay (LOS) and discharge location. Prefracture health status | Partially |  |
| *Valid and Reliable Measurement of PF* | Method of PF measurement is adequately valid and reliable to limit misclassification bias (e.g., may include relevant outside sources of information on measurement properties, also characteristics, such as blind measurement and limited reliance on recall). | Patient characteristics were collected; At the emergency department the participant was given a questionnaire including a pre-paid return envelope. | Yes |  |
|  | Continuous variables are reported or appropriate cut-points (i.e., not data-dependent) are used. | Not described | No |  |
| *Method and Setting of PF Measurement* | The method and setting of measurement of PF is the same for all study participants. | Patient characteristics were collected for all patients | Yes |  |
| *Proportion of data on PF available for analysis* | Adequate proportion of the study sample has complete data for PF variable. | Not described | No |  |
| *Method used for missing data* | Appropriate methods of imputation are used for missing 'PF' data. | Missing items of the HADS were first imputed with individual subscale means according to the half-rule (at least half of the items were answered) [33]. Missing baseline characteristics and missing sum scores in HADS and IES were imputed according to multiple imputation, using the multivariate imputation by chained equations (MICE) procedure | Yes |  |
| **PF Measurement Summary** | ***PF* is adequately measured in study participants to sufficiently limit potential bias.** |  |  | Low |
|  |  |  |  |  |
| **4. Outcome Measurement** | **Goal: To judge the risk of bias related to the measurement of outcome (differential measurement of outcome related to the baseline level of PF).** |  |  |  |
| *Definition of the Outcome* | A clear definition of outcome is provided, including duration of follow-up and level and extent of the outcome construct. | Not described | No |  |
| *Valid and Reliable Measurement of Outcome* | The method of outcome measurement used is adequately valid and reliable to limit misclassification bias (e.g., may include relevant outside sources of information on measurement properties, also characteristics, such as blind measurement and confirmation of outcome with valid and reliable test). | Symptoms of anxiety and depression were measured with the  Hospital Anxiety Depression Scale (HADS). The HADS is internationally known to be a reliable and valid instrument for screening for symptoms of anxiety and depression in a hospitalized older population | Yes |  |
| *Method and Setting of Outcome Measurement* | The method and setting of outcome measurement is the same for all study participants. | At the emergency department the participant was given a questionnaire including a pre-paid return envelope. | Yes |  |
| **Outcome Measurement Summary** | ***Outcome of interest* is adequately measured in study participants to sufficiently limit potential bias.** |  |  | Low |
|  |  |  |  |  |
| **5. Study Confounding** | **Goal: To judge the risk of bias due to confounding (i.e. the effect of PF is distorted by another factor that is related to PF and outcome).** |  |  |  |
| *Important Confounders Measured* | All-important confounders, including treatments (key variables in conceptual model), are measured. | We adjusted for confounding variables in our mixed model analyses. | No |  |
| *Definition of the confounding factor* | Clear definitions of the important confounders measured are provided (e.g., including dose, level, and duration of exposures). | Not described | No |  |
| *Valid and Reliable Measurement of Confounders* | Measurement of all important confounders is adequately valid and reliable (e.g., may include relevant outside sources of information on measurement properties, also characteristics, such as blind measurement and limited reliance on recall). | Not described | No |  |
| *Method and Setting of Confounding Measurement* | The method and setting of confounding measurement are the same for all study participants. | Not described | No |  |
| *Method used for missing data* | Appropriate methods are used if imputation is used for missing confounder data. | Not described | No |  |
| *Appropriate Accounting for Confounding* | Important potential confounders are accounted for in the study design (e.g., matching for key variables, stratification, or initial assembly of comparable groups). | Not described | No |  |
|  | Important potential confounders are accounted for in the analysis (i.e., appropriate adjustment). | Not described | No |  |
| **Study Confounding Summary** | **Important potential confounders are appropriately accounted for, limiting potential bias with respect to the relationship between *PF* and *outcome*.** |  |  | High |
|  |  |  |  |  |
| **6. Statistical Analysis and Reporting** | **Goal: To judge the risk of bias related to the statistical analysis and presentation of results.** |  |  |  |
| *Presentation of analytical strategy* | There is sufficient presentation of data to assess the adequacy of the analysis. | Tables 1-3 | Yes |  |
| *Model development strategy* | The strategy for model building (i.e., inclusion of variables in the statistical model) is appropriate and is based on a conceptual framework or model. | Odds ratios (OR) for the prognostic factors on average following 1 year after hip fracture were calculated in a multivariable logistic mixed model, adjusted for prognostic factors. We performed two multivariable adjusted analyses | Yes |  |
|  | The selected statistical model is adequate for the design of the study. | See above | Yes |  |
| *Reporting of results* | There is no selective reporting of results. | Table 4 | Yes |  |
| **Statistical Analysis and Presentation Summary** | **The statistical analysis is appropriate for the design of the study, limiting potential for presentation of invalid or spurious results.** |  |  | Low |
|  |  |  |  |  |
| Modified from: Hayden JA, Côté P, Bombardier C. Evaluation of the Quality of Prognosis Studies in Systematic Reviews. Annals of Internal Medicine. 2006;144:427-437. | | |  |  |

| **QUIPS Risk of Bias Assessment Instrument for Prognostic Factor Studies** | | |  |  |
| --- | --- | --- | --- | --- |
| **Author and year of publication** | Lai, 2013, Australia |  |  |  |
| **Study identifier** |  |  |  |  |
| **Reviewer** | RMC and GM |  |  |  |
|  |  |  |  |  |
| **Biases** | **Issues to consider for judging overall rating of "Risk of bias"** | **Study Methods & Comments** | **Rating of reporting** | **Rating of "Risk of bias"** |
| Instructions to assess the risk of each potential bias: | These issues will guide your thinking and judgment about the overall risk of bias within each of the 6 domains. Some 'issues' may not be relevant to the specific study or the review research question. These issues are taken together to inform the overall judgment of potential bias for each of the 6 domains. | Provide comments or text exerpts in the white boxes below, as necessary, to facilitate the consensus process that will follow. | Rate the adequacy of reporting as yes, partial, no or unsure. | Rate potential risk of bias for each of the 6 domains as High, Moderate, or Low considering all relevant issues |
| **1. Study Participation** | **Goal: To judge the risk of selection bias (likelihood that relationship between *PF* and *outcome* is different for participants and eligible non-participants).** |  |  |  |
| *Source of target population* | The source population or population of interest is adequately described | A cross-sectional study was conducted on 1127 hip fracture survivors. Patients were aged 50 years or older and received emergency surgery for hip fracture | Yes |  |
| *Method used to identify population* | The sampling frame and recruitment are adequately described, including methods to identify the sample sufficient to limit potential bias (number and type used, e.g., referral patterns in health care) | Not described | No |  |
| *Recruitment period* | Period of recruitment is adequately described | From 2005 to 2008 | Yes |  |
| *Place of recruitment* | Place of recruitment (setting and geographic location) are adequately described | An orthogeriatric unit at Royal Perth Hospital in Western Australia | Yes |  |
| *Inclusion and exclusion criteria* | Inclusion and exclusion criteria are adequately described (e.g., including explicit diagnostic criteria or “zero time” description). | Patients were excluded if they were unable to complete assessments using the GDS. Most exclusions were due to severe cognitive impairment or delirium with poor recovery within the first two weeks of admission. | Partially |  |
| *Adequate study participation* | There is adequate participation in the study by eligible individuals | A total of 1136 patients aged 50 years or older received an  operation for a hip fracture during the study period. GDS-15 was  completed in 1127 patients. All patients who had completed the GDS-15 were included in the analysis | Yes |  |
| *Baseline characteristics* | The baseline study sample (i.e., individuals entering the study) is adequately described | GDS-15 was completed in 1127 patients with 809 female and 318 male patients. Mean GDS-15 score was similar in women and men (2.21±1.74 v 2.17±1.77, P>0.05).  No Table 1 baseline characteristics given | Partially |  |
| **Summary Study participation** | **The study sample represents the population of interest on key characteristics, sufficient to limit potential bias of the observed relationship between PF and outcome.** |  |  | High |
|  |  |  |  |  |
| **2. Study Attrition** | **Goal: To judge the risk of attrition bias (likelihood that relationship between *PF* and *outcome* are different for completing and non-completing participants).** |  |  |  |
| *Proportion of baseline sample available for analysis* | Response rate (i.e., proportion of study sample completing the study and providing outcome data) is adequate. | All patients who had completed the GDS-15 were included in the analysis | Yes |  |
| *Attempts to collect information on participants who dropped out* | Attempts to collect information on participants who dropped out of the study are described. | N/A | N/A |  |
| *Reasons and potential impact of subjects lost to follow-up* | Reasons for loss to follow-up are provided. | N/A | N/A |  |
| *Outcome and prognostic factor information on those lost to follow-up* | Participants lost to follow-up are adequately described | N/A | N/A |  |
|  | There are no important differences between key characteristics and outcomes in participants who completed the study and those who did not. | N/A | N/A |  |
| **Study Attrition Summary** | **Loss to follow-up (from baseline sample to study population analyzed) is not associated with key characteristics (i.e., the study data adequately represent the sample) sufficient to limit potential bias to the observed relationship between PF and outcome.** |  |  | Low |
|  |  |  |  |  |
| **3. Prognostic Factor Measurement** | **Goal: To judge the risk of measurement bias related to how PF was measured (differential measurement of PF related to the level of outcome).** |  |  |  |
| *Definition of the PF* | A clear definition or description of 'PF' is provided (e.g., including dose, level, duration of exposure, and clear specification of the method of measurement). | Age, gender, pre-fracture residence, mobility, the American Society of  Anesthesiologists (ASA) physical status score, pre-existing dementia  and perioperative delirium. | Yes |  |
| *Valid and Reliable Measurement of PF* | Method of PF measurement is adequately valid and reliable to limit misclassification bias (e.g., may include relevant outside sources of information on measurement properties, also characteristics, such as blind measurement and limited reliance on recall). | Data were obtained from an administrative dataset, which  was prospectively collected and maintained | Partial |  |
|  | Continuous variables are reported or appropriate cut-points (i.e., not data-dependent) are used. | Not described | No |  |
| *Method and Setting of PF Measurement* | The method and setting of measurement of PF is the same for all study participants. | Not described | No |  |
| *Proportion of data on PF available for analysis* | Adequate proportion of the study sample has complete data for PF variable. | All patients who had completed the GDS-15 were included in the analysis | Yes |  |
| *Method used for missing data* | Appropriate methods of imputation are used for missing 'PF' data. | N/A | N/A |  |
| **PF Measurement Summary** | ***PF* is adequately measured in study participants to sufficiently limit potential bias.** |  |  | Moderate |
|  |  |  |  |  |
| **4. Outcome Measurement** | **Goal: To judge the risk of bias related to the measurement of outcome (differential measurement of outcome related to the baseline level of PF).** |  |  |  |
| *Definition of the Outcome* | A clear definition of outcome is provided, including duration of follow-up and level and extent of the outcome construct. | Not described | No |  |
| *Valid and Reliable Measurement of Outcome* | The method of outcome measurement used is adequately valid and reliable to limit misclassification bias (e.g., may include relevant outside sources of information on measurement properties, also characteristics, such as blind measurement and confirmation of outcome with valid and reliable test). | GDS-15 is a validated 15-item self-report instrument designed to evaluate symptomatology of depression in older patients | Yes |  |
| *Method and Setting of Outcome Measurement* | The method and setting of outcome measurement is the same for all study participants. | Not described | No |  |
| **Outcome Measurement Summary** | ***Outcome of interest* is adequately measured in study participants to sufficiently limit potential bias.** |  |  | Moderate |
|  |  |  |  |  |
| **5. Study Confounding** | **Goal: To judge the risk of bias due to confounding (i.e. the effect of PF is distorted by another factor that is related to PF and outcome).** |  |  |  |
| *Important Confounders Measured* | All important confounders, including treatments (key variables in conceptual model), are measured. | Linear regression model was used to adjust for confounder variables. | No |  |
| *Definition of the confounding factor* | Clear definitions of the important confounders measured are provided (e.g., including dose, level, and duration of exposures). | See above | No |  |
| *Valid and Reliable Measurement of Confounders* | Measurement of all important confounders is adequately valid and reliable (e.g., may include relevant outside sources of information on measurement properties, also characteristics, such as blind measurement and limited reliance on recall). | See above | No |  |
| *Method and Setting of Confounding Measurement* | The method and setting of confounding measurement are the same for all study participants. | See above | No |  |
| *Method used for missing data* | Appropriate methods are used if imputation is used for missing confounder data. | Not described | No |  |
| *Appropriate Accounting for Confounding* | Important potential confounders are accounted for in the study design (e.g., matching for key variables, stratification, or initial assembly of comparable groups). | Not described | No |  |
|  | Important potential confounders are accounted for in the analysis (i.e., appropriate adjustment). | Not described | No |  |
| **Study Confounding Summary** | **Important potential confounders are appropriately accounted for, limiting potential bias with respect to the relationship between *PF* and *outcome*.** |  |  | High |
|  |  |  |  |  |
| **6. Statistical Analysis and Reporting** | **Goal: To judge the risk of bias related to the statistical analysis and presentation of results.** |  |  |  |
| *Presentation of analytical strategy* | There is sufficient presentation of data to assess the adequacy of the analysis. | Table 2 | Yes |  |
| *Model development strategy* | The strategy for model building (i.e., inclusion of variables in the statistical model) is appropriate and is based on a conceptual framework or model. | Univariate associations were examined between age and GDS. Linear regression model was used to adjust for confounder variables. | Partial |  |
|  | The selected statistical model is adequate for the design of the study. | See above | Yes |  |
| *Reporting of results* | There is no selective reporting of results. | Table 2 | Yes |  |
| **Statistical Analysis and Presentation Summary** | **The statistical analysis is appropriate for the design of the study, limiting potential for presentation of invalid or spurious results.** |  |  | Low |
|  |  |  |  |  |
| Modified from: Hayden JA, Côté P, Bombardier C. Evaluation of the Quality of Prognosis Studies in Systematic Reviews. Annals of Internal Medicine. 2006;144:427-437. | | |  |  |

| **QUIPS Risk of Bias Assessment Instrument for Prognostic Factor Studies** | | |  |  |
| --- | --- | --- | --- | --- |
| **Author and year of publication** | Voshaar, 2007, the Netherlands |  |  |  |
| **Study identifier** |  |  |  |  |
| **Reviewer** | RMC and GM |  |  |  |
|  |  |  |  |  |
| **Biases** | **Issues to consider for judging overall rating of "Risk of bias"** | **Study Methods & Comments** | **Rating of reporting** | **Rating of "Risk of bias"** |
| Instructions to assess the risk of each potential bias: | These issues will guide your thinking and judgment about the overall risk of bias within each of the 6 domains. Some 'issues' may not be relevant to the specific study or the review research question. These issues are taken together to inform the overall judgment of potential bias for each of the 6 domains. | Provide comments or text exerpts in the white boxes below, as necessary, to facilitate the consensus process that will follow. | Rate the adequacy of reporting as yes, partial, no or unsure. | Rate potential risk of bias for each of the 6 domains as High, Moderate, or Low considering all relevant issues |
| **1. Study Participation** | **Goal: To judge the risk of selection bias (likelihood that relationship between *PF* and *outcome* is different for participants and eligible non-participants).** |  |  |  |
| *Source of target population* | The source population or population of interest is adequately described | Every person over the age of 60 who had undergone surgery for a fractured neck of femur | Yes |  |
| *Method used to identify population* | The sampling frame and recruitment are adequately described, including methods to identify the sample sufficient to limit potential bias (number and type used, e.g., referral patterns in health care) | During the recruitment period every person over the age of 60 who had undergone surgery for a fractured neck of femur was within two weeks of operation assessed for study entry | Yes |  |
| *Recruitment period* | Period of recruitment is adequately described | Not described | No |  |
| *Place of recruitment* | Place of recruitment (setting and geographic location) are adequately described | Four orthopaedic units in Manchester, England | Yes |  |
| *Inclusion and exclusion criteria* | Inclusion and exclusion criteria are adequately described (e.g., including explicit diagnostic criteria or “zero time” description). | Patients scoring 6 or less on the 15-item GDS were considered eligible. Exclusion criteria were significant cognitive impairment (defined as a score of less than 18 on the MMSE), significant deafness, severe physical illnesses, unable to speak English and living more than 30 miles outside Manchester | Yes |  |
| *Adequate study participation* | There is adequate participation in the study by eligible individuals | Figure 1 shows patient recruitment. Of 139 patients who gave informed consent, 111 (80%) participated in at least one follow up assessment and were included in the main analyses | Yes |  |
| *Baseline characteristics* | The baseline study sample (i.e., individuals entering the study) is adequately described | Table 2; baseline characteristics | Yes |  |
| **Summary Study participation** | **The study sample represents the population of interest on key characteristics, sufficient to limit potential bias of the observed relationship between PF and outcome.** |  |  | Low |
|  |  |  |  |  |
| **2. Study Attrition** | **Goal: To judge the risk of attrition bias (likelihood that relationship between *PF* and *outcome* are different for completing and non-completing participants).** |  |  |  |
| *Proportion of baseline sample available for analysis* | Response rate (i.e., proportion of study sample completing the study and providing outcome data) is adequate. | Of 139 patients who gave informed consent, 111 (80%) participated in at least one follow up assessment and were included in the main analyses | Yes |  |
| *Attempts to collect information on participants who dropped out* | Attempts to collect information on participants who dropped out of the study are described. | Not described | No |  |
| *Reasons and potential impact of subjects lost to follow-up* | Reasons for loss to follow-up are provided. | Yes, Table 1 | Yes |  |
| *Outcome and prognostic factor information on those lost to follow-up* | Participants lost to follow-up are adequately described for key characteristics. | Baseline characteristics between study participants and dropouts were similar, except for the proportion of patients reporting pain in the hip fracture leg that was significantly less in dropouts compared to study participants (10 of 25 patients, 40% versus 76 of 111, 69%) | Partially |  |
|  | There are no important differences between key characteristics and outcomes in participants who completed the study and those who did not. | See above | Yes |  |
| **Study Attrition Summary** | **Loss to follow-up (from baseline sample to study population analyzed) is not associated with key characteristics (i.e., the study data adequately represent the sample) sufficient to limit potential bias to the observed relationship between PF and outcome.** |  |  | Low |
|  |  |  |  |  |
| **3. Prognostic Factor Measurement** | **Goal: To judge the risk of measurement bias related to how PF was measured (differential measurement of PF related to the level of outcome).** |  |  |  |
| *Definition of the PF* | A clear definition or description of 'PF' is provided (e.g., including dose, level, duration of exposure, and clear specification of the method of measurement). | Not explicitly stated; Results section states that the incident of depression was related to the presence of minimal symptoms of depression, anxiety, pain and less cognitive functioning at baseline, the premorbid level of mobility and a history of (treated) depression | No |  |
| *Valid and Reliable Measurement of PF* | Method of PF measurement is adequately valid and reliable to limit misclassification bias (e.g., may include relevant outside sources of information on measurement properties, also characteristics, such as blind measurement and limited reliance on recall). | Pain was measured by the Wong-Baker pain rating scale and the short form McGill Pain questionnaire | Partial |  |
|  | Continuous variables are reported or appropriate cut-points (i.e., not data-dependent) are used. | Not described | No |  |
| *Method and Setting of PF Measurement* | The method and setting of measurement of PF is the same for all study participants. | Not described | No |  |
| *Proportion of data on PF available for analysis* | Adequate proportion of the study sample has complete data for PF variable. | Not described | No |  |
| *Method used for missing data* | Appropriate methods of imputation are used for missing 'PF' data. | Other cases with missing data were censored at loss to follow-up or in case of non-missing data at six month follow-up | Partial |  |
| **PF Measurement Summary** | ***PF* is adequately measured in study participants to sufficiently limit potential bias.** |  |  | High |
|  |  |  |  |  |
| **4. Outcome Measurement** | **Goal: To judge the risk of bias related to the measurement of outcome (differential measurement of outcome related to the baseline level of PF).** |  |  |  |
| *Definition of the Outcome* | A clear definition of outcome is provided, including duration of follow-up and level and extent of the outcome construct. | Not described | No |  |
| *Valid and Reliable Measurement of Outcome* | The method of outcome measurement used is adequately valid and reliable to limit misclassification bias (e.g., may include relevant outside sources of information on measurement properties, also characteristics, such as blind measurement and confirmation of outcome with valid and reliable test). | Depression was measured by the 15-item GDS. In addition, we included the MADRS and the HADS | Partial |  |
| *Method and Setting of Outcome Measurement* | The method and setting of outcome measurement is the same for all study participants. | All measurements were carried out at baseline, six weeks, three months and six months | Partial |  |
| **Outcome Measurement Summary** | ***Outcome of interest* is adequately measured in study participants to sufficiently limit potential bias.** |  |  | Moderate |
|  |  |  |  |  |
| **5. Study Confounding** | **Goal: To judge the risk of bias due to confounding (i.e. the effect of PF is distorted by another factor that is related to PF and outcome).** |  |  |  |
| *Important Confounders Measured* | All important confounders, including treatments (key variables in conceptual model), are measured. | Not described | No |  |
| *Definition of the confounding factor* | Clear definitions of the important confounders measured are provided (e.g., including dose, level, and duration of exposures). | Not described | No |  |
| *Valid and Reliable Measurement of Confounders* | Measurement of all important confounders is adequately valid and reliable (e.g., may include relevant outside sources of information on measurement properties, also characteristics, such as blind measurement and limited reliance on recall). | Not described | No |  |
| *Method and Setting of Confounding Measurement* | The method and setting of confounding measurement are the same for all study participants. | Not described | No |  |
| *Method used for missing data* | Appropriate methods are used if imputation is used for missing confounder data. | Not described | No |  |
| *Appropriate Accounting for Confounding* | Important potential confounders are accounted for in the study design (e.g., matching for key variables, stratification, or initial assembly of comparable groups). | Not described | No |  |
|  | Important potential confounders are accounted for in the analysis (i.e., appropriate adjustment). | Not described | No |  |
| **Study Confounding Summary** | **Important potential confounders are appropriately accounted for, limiting potential bias with respect to the relationship between *PF* and *outcome*.** |  |  | High |
|  |  |  |  |  |
| **6. Statistical Analysis and Reporting** | **Goal: To judge the risk of bias related to the statistical analysis and presentation of results.** |  |  |  |
| *Presentation of analytical strategy* | There is sufficient presentation of data to assess the adequacy of the analysis. | Table 2; all results including baseline characteristics shown in one table | Partial |  |
| *Model development strategy* | The strategy for model building (i.e., inclusion of variables in the statistical model) is appropriate and is based on a conceptual framework or model. | A Cox proportional hazards model was used to identify risk factors associated with the onset of depression | Partial |  |
|  | The selected statistical model is adequate for the design of the study. | See above | Yes |  |
| *Reporting of results* | There is no selective reporting of results. | Unclear, not all prognostic factors listed in main body of text | No |  |
| **Statistical Analysis and Presentation Summary** | **The statistical analysis is appropriate for the design of the study, limiting potential for presentation of invalid or spurious results.** |  |  | Moderate |
|  |  |  |  |  |
| Modified from: Hayden JA, Côté P, Bombardier C. Evaluation of the Quality of Prognosis Studies in Systematic Reviews. Annals of Internal Medicine. 2006;144:427-437. | | |  |  |

| **QUIPS Risk of Bias Assessment Instrument for Prognostic Factor Studies** | | |  |  |
| --- | --- | --- | --- | --- |
| **Author and year of publication** | Lenze, 2005, USA |  |  |  |
| **Study identifier** |  |  |  |  |
| **Reviewer** | RMC and GM |  |  |  |
|  |  |  |  |  |
| **Biases** | **Issues to consider for judging overall rating of "Risk of bias"** | **Study Methods & Comments** | **Rating of reporting** | **Rating of "Risk of bias"** |
| Instructions to assess the risk of each potential bias: | These issues will guide your thinking and judgment about the overall risk of bias within each of the 6 domains. Some 'issues' may not be relevant to the specific study or the review research question. These issues are taken together to inform the overall judgment of potential bias for each of the 6 domains. | Provide comments or text exerpts in the white boxes below, as necessary, to facilitate the consensus process that will follow. | Rate the adequacy of reporting as yes, partial, no or unsure. | Rate potential risk of bias for each of the 6 domains as High, Moderate, or Low considering all relevant issues |
| **1. Study Participation** | **Goal: To judge the risk of selection bias (likelihood that relationship between *PF* and *outcome* is different for participants and eligible non-participants).** |  |  |  |
| *Source of target population* | The source population or population of interest is adequately described | Admissions to an acute care hospital who had a primary diagnosis of hip fracture. All subjects underwent surgical repair of their hip fracture | Yes |  |
| *Method used to identify population* | The sampling frame and recruitment are adequately described, including methods to identify the sample sufficient to limit potential bias (number and type used, e.g., referral patterns in health care) | Approached consecutive admissions to an acute care hospital who had a primary diagnosis of hip fracture. | Yes |  |
| *Recruitment period* | Period of recruitment is adequately described | March 2002 to September 2003 | Yes |  |
| *Place of recruitment* | Place of recruitment (setting and geographic location) are adequately described | Not described | No |  |
| *Inclusion and exclusion criteria* | Inclusion and exclusion criteria are adequately described (e.g., including explicit diagnostic criteria or “zero time” description). | Subjects were age 60 or older, able to sign informed consent and free of metastatic cancer | Yes |  |
| *Adequate study participation* | There is adequate participation in the study by eligible individuals | Recruited 23 hip fracture subjects (30 subjects were approached, 6 refused and 1 had unsuccessful genotyping for serum) | Yes |  |
| *Baseline characteristics* | The baseline study sample (i.e., individuals entering the study) is adequately described | Table 1 | Yes |  |
| **Summary Study participation** | **The study sample represents the population of interest on key characteristics, sufficient to limit potential bias of the observed relationship between PF and outcome.** |  |  | Low |
|  |  |  |  |  |
| **2. Study Attrition** | **Goal: To judge the risk of attrition bias (likelihood that relationship between *PF* and *outcome* are different for completing and non-completing participants).** |  |  |  |
| *Proportion of baseline sample available for analysis* | Response rate (i.e., proportion of study sample completing the study and providing outcome data) is adequate. | Recruited 23 hip fracture subjects (30 subjects were approached, 6 refused and 1 had unsuccessful genotyping for serum) | Yes |  |
| *Attempts to collect information on participants who dropped out* | Attempts to collect information on participants who dropped out of the study are described. | N/A | N/A |  |
| *Reasons and potential impact of subjects lost to follow-up* | Reasons for loss to follow-up are provided. | N/A | N/A |  |
| *Outcome and prognostic factor information on those lost to follow-up* | Participants lost to follow-up are adequately described | N/A | N/A |  |
|  | There are no important differences between key characteristics and outcomes in participants who completed the study and those who did not. | N/A | N/A |  |
| **Study Attrition Summary** | **Loss to follow-up (from baseline sample to study population analyzed) is not associated with key characteristics (i.e., the study data adequately represent the sample) sufficient to limit potential bias to the observed relationship between PF and outcome.** |  |  | Low |
|  |  |  |  |  |
| **3. Prognostic Factor Measurement** | **Goal: To judge the risk of measurement bias related to how PF was measured (differential measurement of PF related to the level of outcome).** |  |  |  |
| *Definition of the PF* | A clear definition or description of 'PF' is provided (e.g., including dose, level, duration of exposure, and clear specification of the method of measurement). | 5-HTTLPR genotype | Yes |  |
| *Valid and Reliable Measurement of PF* | Method of PF measurement is adequately valid and reliable to limit misclassification bias (e.g., may include relevant outside sources of information on measurement properties, also characteristics, such as blind measurement and limited reliance on recall). | Genotypes section; Obtained a 10 ml sample of blood from each subject for genotyping | Yes |  |
|  | Continuous variables are reported or appropriate cut-points (i.e., not data-dependent) are used. | N/A | N/A |  |
| *Method and Setting of PF Measurement* | The method and setting of measurement of PF is the same for all study participants. | Genotypes section; Obtained a 10 ml sample of blood from each subject for genotyping | Yes |  |
| *Proportion of data on PF available for analysis* | Adequate proportion of the study sample has complete data for PF variable. | 1 participant had unsuccessful genotyping for serum | Yes |  |
| *Method used for missing data* | Appropriate methods of imputation are used for missing 'PF' data. | Not described | No |  |
| **PF Measurement Summary** | ***PF* is adequately measured in study participants to sufficiently limit potential bias.** |  |  | Low |
|  |  |  |  |  |
| **4. Outcome Measurement** | **Goal: To judge the risk of bias related to the measurement of outcome (differential measurement of outcome related to the baseline level of PF).** |  |  |  |
| *Definition of the Outcome* | A clear definition of outcome is provided, including duration of follow-up and level and extent of the outcome construct. | Not described | No |  |
| *Valid and Reliable Measurement of Outcome* | The method of outcome measurement used is adequately valid and reliable to limit misclassification bias (e.g., may include relevant outside sources of information on measurement properties, also characteristics, such as blind measurement and confirmation of outcome with valid and reliable test). | Subjects were assessed at the end of their hospital stay with the PRIME-MD and the 17-item HAM-D. Also used the mood module of the Structured Clinical Interview for DSM-IV Axis I Disorders to ascertain retrospectively whether subjects had a depressive diagnosis, lifetime and immediately before hip fracture | Yes |  |
| *Method and Setting of Outcome Measurement* | The method and setting of outcome measurement is the same for all study participants. | See above | Yes |  |
| **Outcome Measurement Summary** | ***Outcome of interest* is adequately measured in study participants to sufficiently limit potential bias.** |  |  | Low |
|  |  |  |  |  |
| **5. Study Confounding** | **Goal: To judge the risk of bias due to confounding (i.e. the effect of PF is distorted by another factor that is related to PF and outcome).** |  |  |  |
| *Important Confounders Measured* | All important confounders, including treatments (key variables in conceptual model), are measured. | Not explicitly stated; Results section states for one analysis controlled for baseline Functional Independence Measure or Cumulative Illness Rating Scale-Geriatrics scores | Partial |  |
| *Definition of the confounding factor* | Clear definitions of the important confounders measured are provided (e.g., including dose, level, and duration of exposures). | Not explicitly stated | No |  |
| *Valid and Reliable Measurement of Confounders* | Measurement of all important confounders is adequately valid and reliable (e.g., may include relevant outside sources of information on measurement properties, also characteristics, such as blind measurement and limited reliance on recall). | Functional Independence Measure for functional outcome and Cumulative Illness Rating Scale-Geriatrics scores for physical morbidity | Partial |  |
| *Method and Setting of Confounding Measurement* | The method and setting of confounding measurement are the same for all study participants. | Subjects were assessed at the end of their hospital stay and measured functional outcome at baseline (and pre-fracture), 2 weeks and 3 months after fracture | Partial |  |
| *Method used for missing data* | Appropriate methods are used if imputation is used for missing confounder data. | Not described | No |  |
| *Appropriate Accounting for Confounding* | Important potential confounders are accounted for in the study design (e.g., matching for key variables, stratification, or initial assembly of comparable groups). | Not described | No |  |
|  | Important potential confounders are accounted for in the analysis (i.e., appropriate adjustment). | Not described | No |  |
| **Study Confounding Summary** | **Important potential confounders are appropriately accounted for, limiting potential bias with respect to the relationship between *PF* and *outcome*.** |  |  | High |
|  |  |  |  |  |
| **6. Statistical Analysis and Reporting** | **Goal: To judge the risk of bias related to the statistical analysis and presentation of results.** |  |  |  |
| *Presentation of analytical strategy* | There is sufficient presentation of data to assess the adequacy of the analysis. | No table displaying results, only mentioned in text | No |  |
| *Model development strategy* | The strategy for model building (i.e., inclusion of variables in the statistical model) is appropriate and is based on a conceptual framework or model. | A repeated-measures analysis of variance and survival analysis | Partial |  |
|  | The selected statistical model is adequate for the design of the study. | See above | Yes |  |
| *Reporting of results* | There is no selective reporting of results. | The full results are not given in text or displayed in a table | No |  |
| **Statistical Analysis and Presentation Summary** | **The statistical analysis is appropriate for the design of the study, limiting potential for presentation of invalid or spurious results.** |  |  | High |
|  |  |  |  |  |
| Modified from: Hayden JA, Côté P, Bombardier C. Evaluation of the Quality of Prognosis Studies in Systematic Reviews. Annals of Internal Medicine. 2006;144:427-437. | | |  |  |

| **QUIPS Risk of Bias Assessment Instrument for Prognostic Factor Studies** | | |  |  |
| --- | --- | --- | --- | --- |
| **Author and year of publication** | Deng 2005, Taiwan |  |  |  |
| **Study identifier** |  |  |  |  |
| **Reviewer** | RMC and KL |  |  |  |
|  |  |  |  |  |
| **Biases** | **Issues to consider for judging overall rating of "Risk of bias"** | **Study Methods & Comments** | **Rating of reporting** | **Rating of "Risk of bias"** |
| Instructions to assess the risk of each potential bias: | These issues will guide your thinking and judgment about the overall risk of bias within each of the 6 domains. Some 'issues' may not be relevant to the specific study or the review research question. These issues are taken together to inform the overall judgment of potential bias for each of the 6 domains. | Provide comments or text exerpts in the white boxes below, as necessary, to facilitate the consensus process that will follow. | Rate the adequacy of reporting as yes, partial, no or unsure. | Rate potential risk of bias for each of the 6 domains as High, Moderate, or Low considering all relevant issues |
| **1. Study Participation** | **Goal: To judge the risk of selection bias (likelihood that relationship between *PF* and *outcome* is different for participants and eligible non-participants).** |  |  |  |
| *Source of target population* | The source population or population of interest is adequately described | This study is a secondary data analysis | Partially |  |
| *Method used to identify population* | The sampling frame and recruitment are adequately described, including methods to identify the sample sufficient to limit potential bias (number and type used, e.g., referral patterns in health care) | The data was from the cooperation research between Dr XXX and XXX hospital ‘the effect of XXX treatment on prognosis of elderly patients with hip fractures.’ | Partially |  |
| *Recruitment period* | Period of recruitment is adequately described | January 2001 to April 2003 | Yes |  |
| *Place of recruitment* | Place of recruitment (setting and geographic location) are adequately described | The department of trauma in a north medical centre | Partially |  |
| *Inclusion and exclusion criteria* | Inclusion and exclusion criteria are adequately described (e.g., including explicit diagnostic criteria or “zero time” description). | The recruitment requirements are: 1) diagnosed of hip fracture and no other diseases of skeleton, nervous or muscle system. 2) first time hip fracture and no joint replacement surgery before 3) the muscle strength of upper limbs and the healthy lower limb > 4 points 4) >= 60 years old 5) clear consciousness and agree to attend this research. | Yes |  |
| *Adequate study participation* | There is adequate participation in the study by eligible individuals | This study investigated 158 people; the effective sample size is 146 (92.4%). | Yes |  |
| *Baseline characteristics* | The baseline study sample (i.e., individuals entering the study) is adequately described | Table 1; average age is 77.7 years old (SD=7.8, range :60~98), the female are more than the male, more than 90% of the above have no jobs. | Yes |  |
| **Summary Study participation** | **The study sample represents the population of interest on key characteristics, sufficient to limit potential bias of the observed relationship between PF and outcome.** |  |  | Low |
|  |  |  |  |  |
| **2. Study Attrition** | **Goal: To judge the risk of attrition bias (likelihood that relationship between *PF* and *outcome* are different for completing and non-completing participants).** |  |  |  |
| *Proportion of baseline sample available for analysis* | Response rate (i.e., proportion of study sample completing the study and providing outcome data) is adequate. | Not described | No |  |
| *Attempts to collect information on participants who dropped out* | Attempts to collect information on participants who dropped out of the study are described. | Not described | No |  |
| *Reasons and potential impact of subjects lost to follow-up* | Reasons for loss to follow-up are provided. | Not described | No |  |
| *Outcome and prognostic factor information on those lost to follow-up* | Participants lost to follow-up are adequately described | Not described | No |  |
|  | There are no important differences between key characteristics and outcomes in participants who completed the study and those who did not. | Not described | No |  |
| **Study Attrition Summary** | **Loss to follow-up (from baseline sample to study population analyzed) is not associated with key characteristics (i.e., the study data adequately represent the sample) sufficient to limit potential bias to the observed relationship between PF and outcome.** |  |  | High |
|  |  |  |  |  |
| **3. Prognostic Factor Measurement** | **Goal: To judge the risk of measurement bias related to how PF was measured (differential measurement of PF related to the level of outcome).** |  |  |  |
| *Definition of the PF* | A clear definition or description of 'PF' is provided (e.g., including dose, level, duration of exposure, and clear specification of the method of measurement). | Gender, Cognition, Residence status, Prefracture physical function, support system, age, marital status, religion, occupation, diseases before admission | Partial |  |
| *Valid and Reliable Measurement of PF* | Method of PF measurement is adequately valid and reliable to limit misclassification bias (e.g., may include relevant outside sources of information on measurement properties, also characteristics, such as blind measurement and limited reliance on recall). | Gender, Cognition (measured using MMSE-Chinese version), Residence status, Prefracture physical function (measured using the Chinese version of the Barthel Index and Instrumental Activities of Daily Living Scale), support system (measured using the MOS Social support survey)  A questionnaire of basic characteristics also measured age, marital status, religion, occupation, diseases before admission | Partial |  |
|  | Continuous variables are reported or appropriate cut-points (i.e., not data-dependent) are used. | Not described | No |  |
| *Method and Setting of PF Measurement* | The method and setting of measurement of PF is the same for all study participants. | Not described | No |  |
| *Proportion of data on PF available for analysis* | Adequate proportion of the study sample has complete data for PF variable. | Not described | No |  |
| *Method used for missing data* | Appropriate methods of imputation are used for missing 'PF' data. | Not described | No |  |
| **PF Measurement Summary** | ***PF* is adequately measured in study participants to sufficiently limit potential bias.** |  |  | High |
|  |  |  |  |  |
| **4. Outcome Measurement** | **Goal: To judge the risk of bias related to the measurement of outcome (differential measurement of outcome related to the baseline level of PF).** |  |  |  |
| *Definition of the Outcome* | A clear definition of outcome is provided, including duration of follow-up and level and extent of the outcome construct. | Not described | No |  |
| *Valid and Reliable Measurement of Outcome* | The method of outcome measurement used is adequately valid and reliable to limit misclassification bias (e.g., may include relevant outside sources of information on measurement properties, also characteristics, such as blind measurement and confirmation of outcome with valid and reliable test). | Geriatric Depression Scale – Short Form (GDS-SF) | Partially |  |
| *Method and Setting of Outcome Measurement* | The method and setting of outcome measurement is the same for all study participants. | Not described | No |  |
| **Outcome Measurement Summary** | ***Outcome of interest* is adequately measured in study participants to sufficiently limit potential bias.** |  |  | High |
|  |  |  |  |  |
| **5. Study Confounding** | **Goal: To judge the risk of bias due to confounding (i.e. the effect of PF is distorted by another factor that is related to PF and outcome).** |  |  |  |
| *Important Confounders Measured* | All important confounders, including treatments (key variables in conceptual model), are measured. | Not described | No |  |
| *Definition of the confounding factor* | Clear definitions of the important confounders measured are provided (e.g., including dose, level, and duration of exposures). | Not described | No |  |
| *Valid and Reliable Measurement of Confounders* | Measurement of all important confounders is adequately valid and reliable (e.g., may include relevant outside sources of information on measurement properties, also characteristics, such as blind measurement and limited reliance on recall). | Not described | No |  |
| *Method and Setting of Confounding Measurement* | The method and setting of confounding measurement are the same for all study participants. | Not described | No |  |
| *Method used for missing data* | Appropriate methods are used if imputation is used for missing confounder data. | Not described | No |  |
| *Appropriate Accounting for Confounding* | Important potential confounders are accounted for in the study design (e.g., matching for key variables, stratification, or initial assembly of comparable groups). | Not described | No |  |
|  | Important potential confounders are accounted for in the analysis (i.e., appropriate adjustment). | Not described | No |  |
| **Study Confounding Summary** | **Important potential confounders are appropriately accounted for, limiting potential bias with respect to the relationship between *PF* and *outcome*.** |  |  | High |
|  |  |  |  |  |
| **6. Statistical Analysis and Reporting** | **Goal: To judge the risk of bias related to the statistical analysis and presentation of results.** |  |  |  |
| *Presentation of analytical strategy* | There is sufficient presentation of data to assess the adequacy of the analysis. | Table 3; whether univariate or multivariate analysis done not stated, no confounding variables stated | Partial |  |
| *Model development strategy* | The strategy for model building (i.e., inclusion of variables in the statistical model) is appropriate and is based on a conceptual framework or model. | Not described | No |  |
|  | The selected statistical model is adequate for the design of the study. | Not described | No |  |
| *Reporting of results* | There is no selective reporting of results. | Not described | No |  |
| **Statistical Analysis and Presentation Summary** | **The statistical analysis is appropriate for the design of the study, limiting potential for presentation of invalid or spurious results.** |  |  | High |
|  |  |  |  |  |
| Modified from: Hayden JA, Côté P, Bombardier C. Evaluation of the Quality of Prognosis Studies in Systematic Reviews. Annals of Internal Medicine. 2006;144:427-437. | | |  |  |
